# What Is the Role of the Environment in the Emergence of Novel Antibiotic Resistance Genes? A Modeling Approach

**DOI:** 10.1021/acs.est.1c02977

**Published:** 2021-11-18

**Authors:** Johan Bengtsson-Palme, Viktor Jonsson, Stefanie Heß

**Affiliations:** † Department of Infectious Diseases, Institute of Biomedicine, The Sahlgrenska Academy, University of Gothenburg, Guldhedsgatan 10, SE-413 46 Gothenburg, Sweden; ‡ Centre for Antibiotic Resistance Research (CARe) at University of Gothenburg, 405 30 Gothenburg, Sweden; § Integrated Science Lab, Department of Physics, Umeå University, SE-901 87 Umeå, Sweden; ∥ Institute of Microbiology, Technische Universität Dresden, Zellescher Weg 20b, 01847 Dresden, Germany

**Keywords:** human and animal health, mobile genetic elements, mobilization, origin of antibiotic resistance genes, pathogenic bacteria

## Abstract

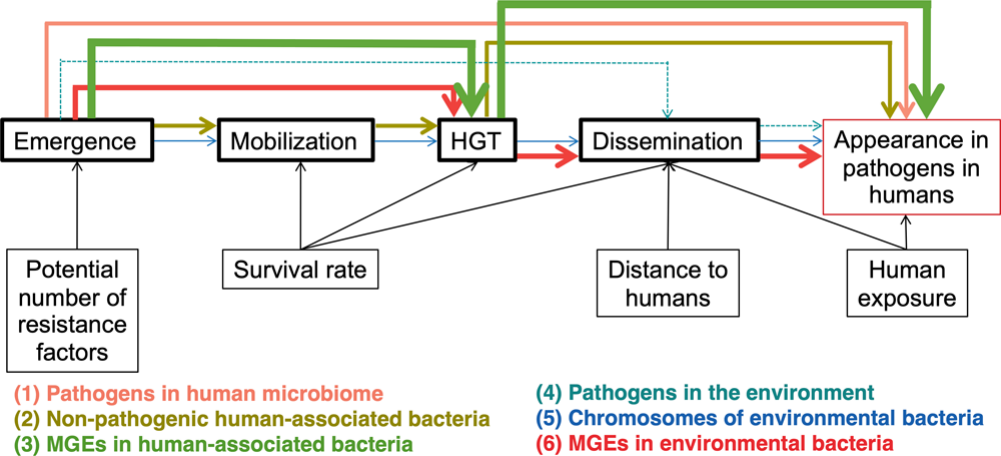

It is generally accepted that intervention strategies to curb antibiotic resistance cannot solely focus on human and veterinary medicine but must also consider environmental settings. While the environment clearly has a role in transmission of resistant bacteria, its role in the emergence of novel antibiotic resistance genes (ARGs) is less clear. It has been suggested that the environment constitutes an enormous recruitment ground for ARGs to pathogens, but its extent is practically unknown. We have constructed a model framework for resistance emergence and used available quantitative data on relevant processes to identify limiting steps in the appearance of ARGs in human pathogens. We found that in a majority of possible scenarios, the environment would only play a minor role in the emergence of novel ARGs. However, the uncertainty is enormous, highlighting an urgent need for more quantitative data. Specifically, more data is most needed on the fitness costs of ARG carriage, the degree of dispersal of resistant bacteria from the environment to humans, and the rates of mobilization and horizontal transfer of ARGs. This type of data is instrumental to determine which processes should be targeted for interventions to curb development and transmission of ARGs in the environment.

## Abstract 2

This work uses modeling to elucidate the role of the environment in the emergence of novel antibiotic resistance genes in pathogens.

## Introduction

Antibiotic resistance is a globally growing health threat, which is projected to take more lives than all forms of cancer combined by 2050 if it cannot be controlled.^1^ Nowadays, there is a general consensus that intervention strategies should consider not only human and veterinary medicine but also take into account the environment, in what is referred to as a one health approach.^2^ While surveillance programs and careful reduction of the use of antibiotics in clinical and agricultural settings started years ago, we are still only starting to understand the role of the environment in the development and dissemination of antibiotic resistance.^3−5^ Monitoring data on the abundance and diversity of antibiotic resistance genes (ARGs) has provided a conceptual understanding of the dispersal routes for ARGs between and within humans, farmed animals and the external environment.^6−8^ We also have a reasonably clear picture of what processes result in recruitment of ARGs from environmental bacteria to human pathogens.^9^ However, much remains to be done in terms of quantifying the relative importance of these processes, which is indispensable knowledge for effective strategies to minimize the spread and development of antibiotic resistance.^10^

It has been proposed that the environment could have three main roles in resistance development.^9^ First, it enables the transfer of ARGs between environmental, human, and animal associated bacteria. Second, it constitutes a reservoir or intermediate habitat for resistant bacteria and ARGs. Finally, it can play a crucial role in the evolution of novel resistance factors, as it provides an arena for selection of resistance combined with an enormous source of genetic diversity from which bacteria can recruit ARGs.^8,9^ In this paper, we have chosen to use the definitions of Bengtsson-Palme *et al.*(9) and consequently defined the “emergence” of an ARG as the event where it first appears in a context in which it provides operational resistance.^11^ Furthermore, a gene is considered to be “mobilized” when it appears on a mobile genetic element (MGE), such as a plasmid, transposon, or integron. Newly emerged ARGs from the environment can subsequently be disseminated into the human and farmed animals’ compartments, where they have the potential to cause severe health threats.

While this conceptual role of the environment is well described, its actual importance in resistance development is so far unknown, largely due to a lack of quantitative data.^10,12^ Particularly, the probabilities of emergence of novel ARGs in the environment and their transfer rates to human pathogens are completely unknown this far.^12^ This means that it becomes very hard to rank risk scenarios and prioritize between possible interventions targeting the emergence of novel ARGs. The primary aim of this study is to identify the steps within this chain of processes, which limit the appearance of ARGs in human pathogens. To address this, we set up a model framework and quantified the dependencies of variables given estimates for upper and lower bounds for the process rates available in the literature. Mathematical models of the spread and evolution of ARGs allow us to better understand the underlying processes and thus make better policy decisions.^13^ Although several models exist that target specific situations, e.g., the selection for resistance in agricultural waste^14^ or the evolution of ARGs in a small population,^15,16^ we here, in contrast, take a comprehensive approach to capture the flow of ARGs to human pathogens throughout the antibiotic era. Finally, we assess how the uncertainty in the available data affects certain parameters in the model, thereby highlighting where the most urgent needs for quantitative data exist.

## Materials and Methods

### Model Design

**Figure 1:**
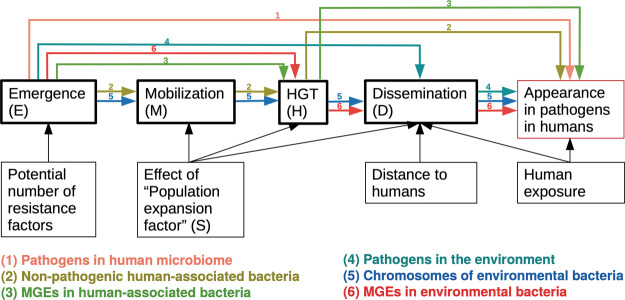
Overview of the model framework. Processes are framed in bold. Arrows display the influence of different parameters on the processes. Colored arrows represent the pathways included in the model (*E*_1_ to *E*_6_).

We have modeled different pathways that can result in recruitment of novel ARGs to pathogens, following previous conceptual model development (Figure 1).^9^ It is likely that ARGs that now are present in human pathogens already pre-existed in bacteria in some setting at the start of the antibiotic era (around 70 years ago) and only needed to be, in some way, transferred to human pathogens.^17−19^ This scenario (the “pre-existing model”) is therefore our main model in this study. To contrast this, we also implemented a second model scenario where we assume that ARGs did not have a resistance function at the start of the modeled time period but needed to first emerge as resistance factors, referred to here as the “emergence model”. This second scenario represents an alternative model for the evolution of ARGs. The measurable endpoint of both the proposed models is a mobilized ARG observed in a human pathogen, which is compared to an actual ARG appearance rate estimated from public databases. Each of the six pathways shown in Figure 1 is expressed as equations (Table 1) with parameters defined in Table 2. The model was implemented in *R* as follows. Each parameter was randomly selected from its log10 transformed range under a uniform distribution. For each set of randomly selected parameters, the result of the equations for the different considered processes was calculated and the total appearance was determined based on the sub-equations according to this formula:
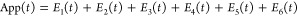
where *E*_1_ to *E*_6_ each represent the contribution of ARGs by a particular process (Figure 1), expressed as the total contributed ARGs over the entire model timeframe, and *t* is the time in days. Each *E_n_* represents one potential pathway for ARGs to end up in human pathogens, given their location 70 years ago. If the modeled total appearance fell within the expected interval, i.e., the number of mobile ARGs present in pathogens predicted by the model approximately matched the observed number of mobile ARGs in pathogens today (700 to 2200 ARGs), the model parameters were saved. This procedure was iterated until 10,000 valid sets of model parameters had been obtained for each modeled time point and set of parameter ranges. The full details on the model implementation are available in the Supporting Information.

**Table 1:** Considered Pathways and the Corresponding Equations in the Two Models^a^

description of pathway	equation (pre-existing model)	equation (emergence model)
appearance directly on a mobile genetic element in a human pathogen	*E*_1_(*t*) = 10^30^ × *Pph* × *Pm* × *E* × *S^t^*	*E*_1_(*t*) = 10^30^ × *Pph* × *Pm* × *E* × *t* × *S*^*t*/2^
appearance in non-pathogenic human bacteria, mobilization, and transfer to human pathogens	*E*_2_(*t*) = 10^30^ × *Ph* × *E* × *MH* × *t* × *S^t^*	*E*_2_(t) = 10^30^ × *Ph* × *E* × *t* × *MH* × *t*/2 × *S^t/2^*
appearance on a mobile genetic element in non-pathogenic human-associated bacteria, and transfer to human pathogens	*E*_3_(*t*) = 10^30^ × *Ph* × *Pm* × *E* × *H* × *t* × *S^t^*	*E*_3_(*t*) = 10^30^ × *Ph* × *Pm* × *E* × *t* × *H* × *t*/2 × *S^t/2^*
appearance in pathogens in the environment and dissemination to humans	*E*_4_(*t*) = 10^30^ × *Pp* × *Pm* × *E* × *S^t^* × *D* × *t*	*E*_4_(*t*) = 10^30^ × *Pp* × *Pm* × *E* × *t* × *S^t/2^* × *D* × *t*/2
appearance in environmental bacteria, mobilization, transfer to pathogens, and dissemination to humans	*E*_5_(*t*) = 10^30^ × *E* × *MH* × *t* × *S^t^* × *D* × *t*/2	*E*_5_(*t*) = 10^30^ × *E* × *t* × *MH* × *t*/2 × *S^t/2^* × *D* × *t*/4
appearance on a mobile genetic element in environmental bacteria, transfer to pathogens, and dissemination to humans	*E*_6_(*t*) = 10^30^ × *Pm* × *E* × *H* × *t* × *S^t^* × *D* × *t*/2	*E*_6_(*t*) = 10^30^ × *Pm* × *E* × *t* × *H* × *t*/2 × *S^t/2^* × *D* × *t*/4
		

a For process and parameter abbreviations, see Table 2; *t* is for time in days; 10^30^ represents the approximate total number of bacteria on earth.

**Table 2:** Estimates for the Respective Process Rates and Parameters Used in the Model

abbreviation	meaning	unit	literature values			model boundaries	
			lower bound	upper bound	median	lower bound	upper bound
*E*	proportion of bacteria in which a given ARG exists at the model start		unknown			10^–30^	1
	or						
	the emergence rate of a novel ARG (in an emergence model)	events/cell/day	unknown			10^–40^	1
*App*	first detected appearance of a novel ARG in a human pathogen in a human	events/day	0.025	0.079	N/A	0.025	0.079
*M*	mobilization of a chromosomally encoded ARG onto a plasmid, transposon or other conjugative element	events/cell/day	difficult to disentangle from horizontal transfer based on current experimental evidence, see *MH*				
*H*	horizontal gene transfer (conjugation)	events/cell/day	2.4 × 10^–10^	5.8 × 10^–2^	3.0 × 10^–3^	10^–11^	10^–1^
*D*	dissemination from the environment to humans	events/cell/day	10^–14^	10^–11^	N/A	10^–15^	10^–10^
*Pph*	fraction of all bacterial cells that are human pathogens and living in/on humans		∼10^–10^ (∼10^13^ bacterial cells per human × ∼10^10^ humans worldwide × ∼10^–3^ bacteria living in humans pathogenic/∼10^30^ bacterial cells in the world)			10^–12^	10^–8^
Ph	fraction of all bacterial cells that live in/on humans of the total bacterial cells in the world		∼10^–7^ (∼10^13^ bacterial cells per human × ∼10^10^ humans worldwide/∼10^30^ bacterial cells in the world)			10^–8^	10^–6^
*MH*	mobilization of a chromosomally encoded ARG and transfer into another strain	events/cell/day	4 × 10^–10^	1.5 × 10^–4^	∼5.67 × 10^–9^	10^–15^	10^–2^^a^
*S*	population expansion rate (1 corresponds to no change in population size)	1/day	unknown			0.9	1.1
*Pm*	probability that a novel ARG emerges on a plasmid		0.002 (∼7% of all bacterial cells carry a conjugative plasmid; most plasmids have ∼50–100 genes, about 3% of the total bacterial genome)			0.0001	0.01
*Pp*	fraction of human pathogenic cells (in all compartments) of bacterial cells in the world		10^–9^, must be larger than *Pph*, but how much larger is unknown			10^–11^	10^–7^

a *MH* is also restricted to be <*H*.

### Defining the Probability Boundaries for Antibiotic Resistance Development

In order to populate the model, measured values for the individual parameters were taken from the literature (Tables S1 and S2), and the upper and lower boundaries were fed into the model (Table 2). For the rates of horizontal gene transfer (HGT), captured by the parameter (*H*), most studies were found for conjugative transfer of genes. Data for both intra- and interspecies transfer of various naturally occurring and artificially constructed plasmids were available. These have been included as a first approximation in the model, bearing in mind that this is laboratory data and that their transferability to the respective ecosystems has been minimally studied so far. At this time, we have limited knowledge concerning the role of transduction and transformation for the distribution of ARGs in the respective ecosystems, although recent studies have suggested that they may be important in the transfer of ARGs between bacteria.^20^ Due to the lack of quantitative data, these two processes were not considered for the estimation of the parameter *H*.

Unfortunately, there are no direct measurements for the mobilization parameter (*M*) that have been obtained independent of HGT. Most studies have the following design in common:^21−25^ the resistance encoded by the donor is not mobile and can only be detected in the recipient once it has been mobilized and transferred. Thus, we do not have a measure of the *M* parameter alone. Instead, we have used the combined parameter *MH* (mobilization and HGT) for which experimental data could be obtained from these studies.

To get an estimate for the dissemination of ARGs from the environment to humans (*D*), the number of bacteria eaten per day was used as a proxy.^26^ In that study, the authors counted the number of colony forming units of meals for three different diet types. However, other transfer routes are also conceivable, e.g., the absorption of bacteria by swallowing water while bathing.^27,28^ Such dissemination pathways can be of decisive importance when considering individual systems. However, they are not explicitly further considered in this model, which is intentionally kept general, as we aim to accommodate all possible dispersal scenarios in one single parameter.

Finally, some parameters were calculated based on the estimates of the number of humans on earth (∼8 billion), the number of bacterial cells in/on the human body (3.8 × 10^13^),^29^ and the total number of bacterial cells on earth (in the order of 10^30^).^30^ These values were used to define ranges for *Pph*, *Ph*, *Pp*, and *E* (Table 2). We also estimate a typical bacterial genome to contain 1500 to 7500 genes,^31^ that around 7% of bacterial cells carry a conjugable plasmid, and that a typical plasmid carries 50–100 genes,^32^ which was used to constrain the Pm parameter. The *S* parameter represents the overall fitness impact of carrying an ARG and implicitly also involves the survival rate for a bacterium carrying an ARG, relative to a non-carrier. *S* is defined as the relative population expansion rate per day in the model, so when *S* is equal to 1, the average ARG would have no impact on the expansion of the population, i.e., the average ARG would overall be fitness neutral.

## Results and Discussion

### Many ARGs Were Likely Present in Human-Associated Bacteria at the Beginning of the Antibiotic Era

**Figure 2:**
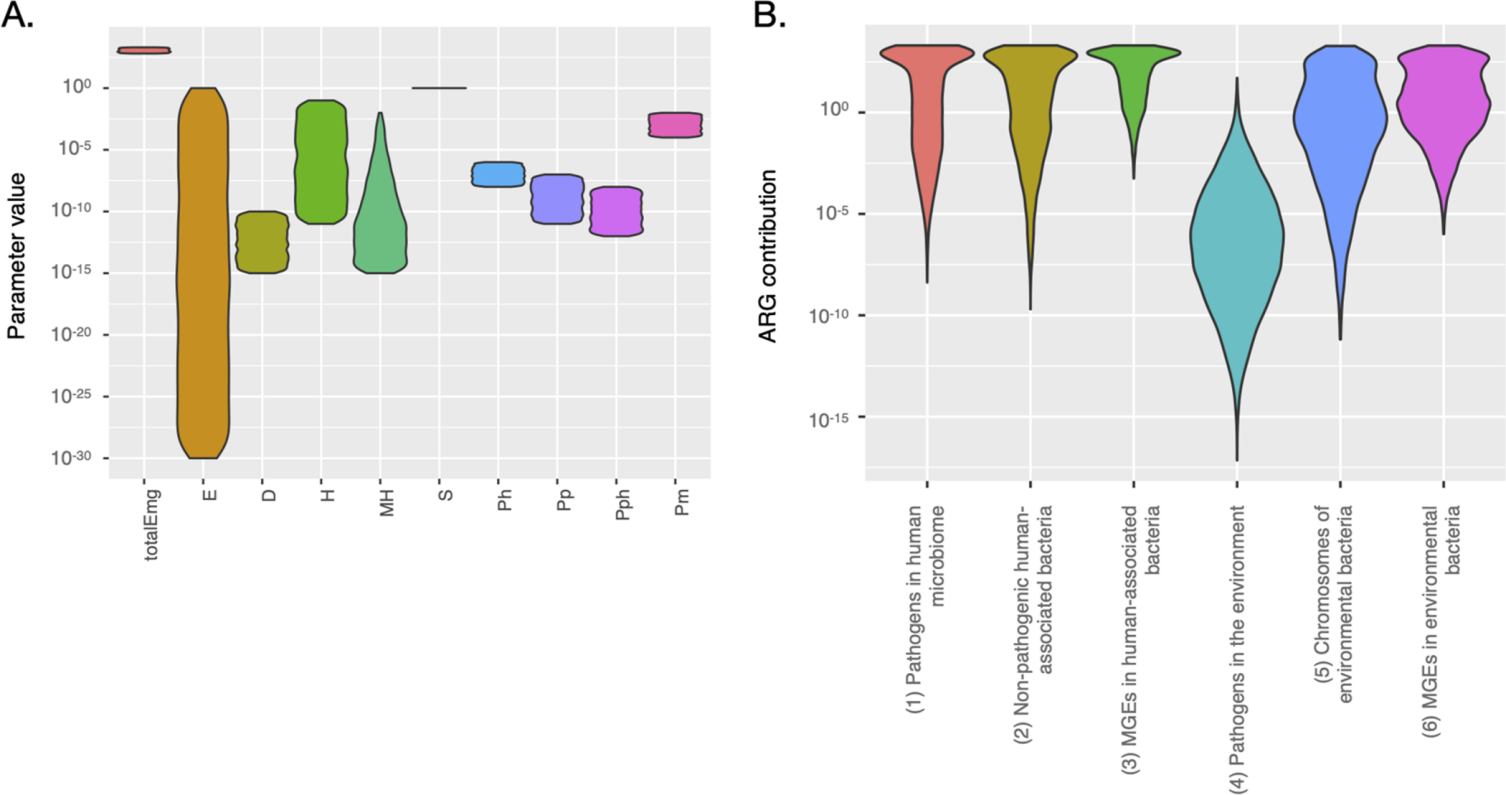
Valid parameter ranges (A) and process rates (B) for the main model after 70 years of simulated time. The range of *S* extends from around 0.99 to 1.002. Since every parameter has its own definition, the parameter values in (A) have slightly different meanings (see Table 2).

**Figure 3:**
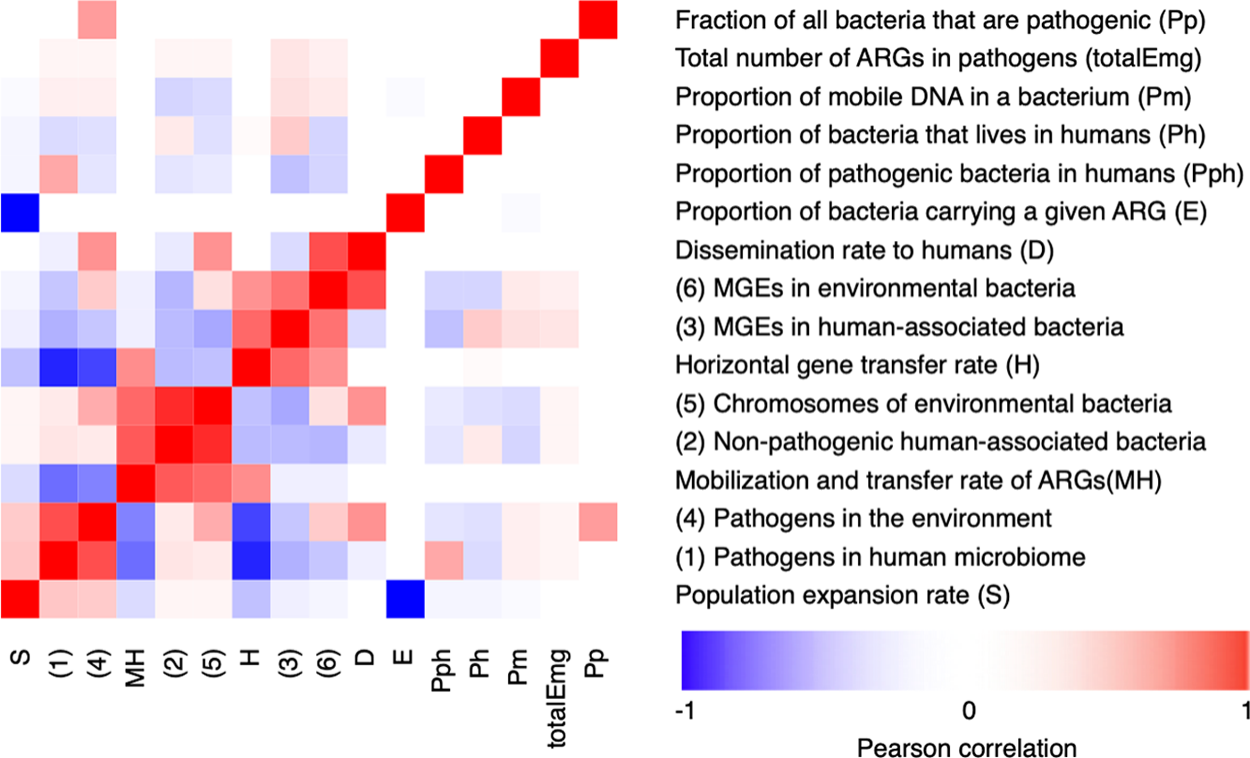
Correlations between the variables in the main model after 70 years of simulated time. Blue colors represent negative correlation values, red colors represent positive associations, and white indicates unrelated parameters. totalEmg represents the total number of ARGs that have emerged on MGEs in human pathogens.

It is clear from the model results that for many parameters, the permitted range of possible values is huge (Figure 2A). Even for intuitively ridiculous models such as the assumption that all bacteria originally carried all ARGs (*E* = 1), there seem to be valid model scenarios. However, it is clear that in those cases, this would have to be compensated by the vast majority of ARGs having a strongly negative effect on fitness, as evidenced by the strong negative correlation between *E* and *S* (Figure 3 and Figure S1). As the model results are highly dependent on certain parameters, it is difficult to say what the typical result of the model would be. However, assuming that the dispersal parameter *D* is in the range between 10^–12^ and 10^–11^, the most likely origins for the currently circulating ARGs at the start of the antibiotic era would be MGEs in human-associated bacteria (65.6%) and MGEs in environmental bacteria (28.4%; Table 3). These two origins are substantially more likely than ARGs having been present in pathogens at the start of the antibiotic era (3.07%), coming from the chromosomes of non-pathogenic human-associated bacteria (2.03%) or from the chromosomes of environmental bacteria (0.883%). In almost all runs of the model, the likelihood of ARGs originating from pathogenic bacteria in the environment is less than 0.00001% (Table 3). However, the possible ranges for all the other five processes are highly overlapping (Figure 2B).

**Table 3:** Dependency of the Modeled Processes on the *D* Parameter

origin	*D*: 10^–15^ to 10^–14^	*D*: 10^–14^ to 10^–13^	*D*: 10^–13^ to 10^–12^	*D*: 10^–12^ to 10^–11^	*D*: 10^–11^ to 10^–10^
*E*_1_: pathogens in the human microbiome	3.31%	3.74%	3.88%	3.07%	0.733%
*E*_2_: non-pathogenic human-associated bacteria	2.81%	3.19%	2.63%	2.03%	0.530%
*E*_3_: MGEs in human-associated bacteria	93.8%	92.7%	89.9%	65.6%	18.9%
*E*_4_: pathogenic bacteria in the environment	<0.00001%	<0.00001%	<0.00001%	<0.00001%	0.00001%
*E*_5_: chromosomes of environmental bacteria	0.00116%	0.123%	0.103%	0.883%	2.18%
*E*_6_: MGEs in the environment	0.0389%	0.358%	3.50%	28.4%	77.7%

Furthermore, the interdependencies between the process rates and some of the individual parameters are, in many cases, fairly high. For example, the likelihood of an ARG originating from the chromosomes of environmental bacteria or from non-pathogenic human-associated bacteria is strongly correlated to the value of *MH* (Figure 3). Similarly, the proportion of ARGs that already existed in pathogens in humans to begin with is, unsurprisingly, dependent on the proportion of all bacteria that are human pathogens (*Pph*), and the likelihood of it originating on an MGE, either in a human-associated bacterium or in the environment, is strongly dependent on the rate of HGT (*H*). Importantly, though, the number of bacteria carrying a certain ARG at the start of the modeling time (*E*) is almost entirely dependent on the fitness cost or advantage associated with the gene (*S*), highlighting that selection for ARGs is by far the most important process in ARG ecology.

### Dispersal Limitation Constrains the Role of the Environment in ARG Emergence

The parameter that largely controls whether the environment is likely to be a significant contributor to ARGs in pathogens is *D* (Figure 3). If the environment played a major role in the emergence of the nowadays known ARGs in human pathogens, as has been argued by many authors,^4,17,33,34^ dissemination from the environment to humans (*D*) as well as the mobilization and transfer rates (*MH*) of ARGs need to be fairly high. Importantly, the data on dispersal of genes from environmental bacteria to the human population is very limited, particularly for the type of generalized case that we outline in this paper. Due to the lack of data, we have chosen a fairly naive definition of dispersal for our model, reflecting the type of data that is available. The *D* parameter in the model represents the proportion of environmental bacteria that would be expected to be exposed to a human somewhere in the world during the day. However, it does not account for bacterial exposure through other routes than dietary intake, such as recreational swimming,^27,28^ wounds,^35^ and through direct contact with animals.^34,36,37^ Thus, the definition of *D* in our model does not reflect that the origins of bacteria that the average human is exposed to are not equally distributed across environments. To some extent, we expect this to be compensated by bacterial global dispersal over time, but it still reflects a weakness in our model. That said, although it is feasible that all these activities contribute significant numbers of bacteria, it is unlikely that they would change *D* by several orders of magnitude. However, it may be fair to assume that *D* is in the higher end of the spectrum, i.e., larger than 10^–12^ rather than close to the lower boundary. If *D* would be larger than 10^–11^, environmental bacteria may have contributed around 80% of all currently clinically relevant ARGs (Table 3). The impact of the *D* parameter highlights that determining the actual dispersal rate of ARGs from the environment is crucial to understand the role of the environment in antibiotic resistance development. Comprehensive data on ARG dispersal would not only aid in risk assessment of emergence of novel ARGs but it would also aid in understanding and mitigating the spread of resistant bacteria (and their ARGs) through the environment.^6,8,10,38^

### HGT and Mobilization Determine the Importance of Chromosomal ARGs

It is notable that the three parameters that influence the outcomes of the model the most are *S*, *H*, and *MH* (and indirectly *E*, as it is anticorrelated with *S*; Figure 3). In particular, changing the values of *MH* and *H* shifts the relative importance of the processes and thus the most likely origin of ARGs dramatically (Table S3). Still, these processes are less likely to be bottlenecks in resistance development than selection and dispersal. At high rates of HGT, most ARGs are, according to the model, likely to have originated on MGEs, unless the mobilization rate is also high. Unfortunately, the latter parameter is almost completely unknown (Table 2). Furthermore, since these estimates are all based on mobilization followed by HGT, they do not represent the mobilization rate *per se*, and thus the actual rate may turn out to be outside of the mentioned range. In addition, these rates have been measured using very specific markers that are known to be transferable to plasmids, which may not make them representative for the mobilization rate of ARGs in general. In addition, there is a need to better define the typical rates of HGT between bacteria. Several assays already exist to perform such experiments,^39−42^ but the amount of data available is still insufficient as it has only been generated for a very limited number of conditions, ARGs and mating pairs. Furthermore, most research studies on HGT have dealt with conjugation, while transformation and transduction have been largely neglected, particularly from a quantitative point of view. Still, the two latter processes have been shown to be important drivers of the horizontal transfer of ARGs,^20,43−45^ although their contribution relative to conjugation is unclear. Importantly, this is largely a data generation problem. In contrast, the lack of knowledge of the rate of mobilization of chromosomal genes to MGEs is not only related to insufficient data but also to a lack of appropriate assays to actually measure mobilization of ARGs without involvement of, e.g., HGT. This highlights an urgent need for assays to directly detect mobilization of genes from the chromosomes to MGEs. Still, it should be pointed out that the span of possible outcomes for a given size of *MH*, *H*, or *D* is still very large (Figures S2–S4).

### Stability of the Model Parameters to Changes

**Figure 4:**
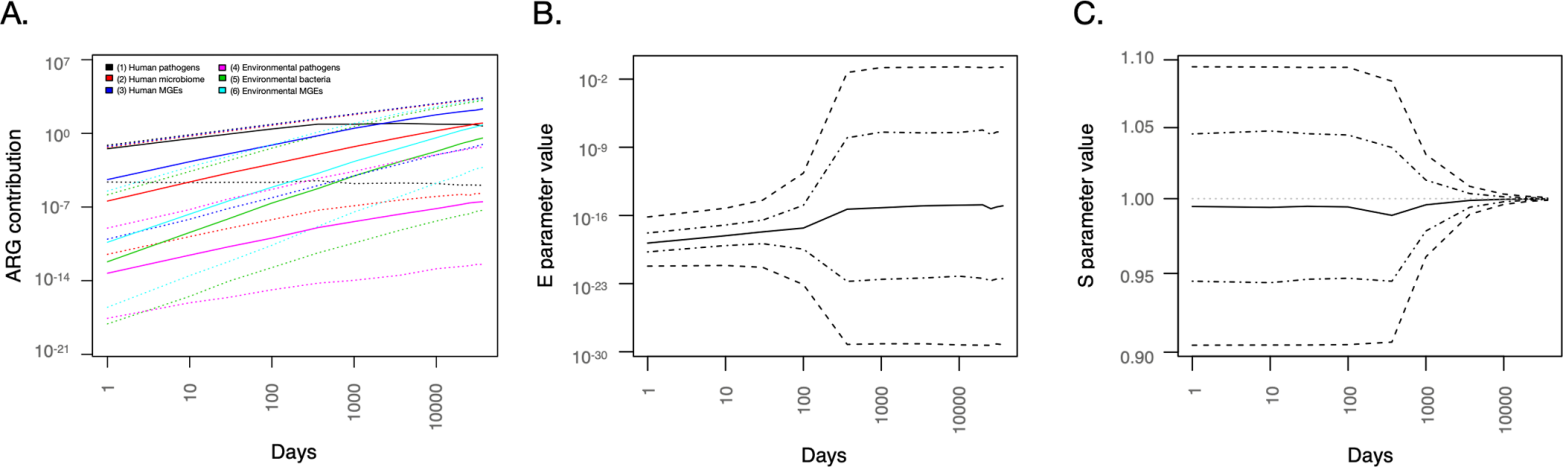
Time dependence of model process rates (A) and key model parameters E (B) and S (C) over the simulated time span from the start of antibiotic use to the emergence of ARGs in human pathogens. ARG contribution is expressed as the number of ARGs originating from each process (maximum of 2200 at 10,000 days) in (A). Dotted lines in (A) and dashed lines in (B) and (C) represent the range in which 95% of the values fall in the simulations. Lines with dots and dashes in (B) and (C) represent the range in which 50% of the values fall in the simulations.

We also investigated how stable the model was over time (i.e., when the number of days was varied in the model; Figure 4). Again, the strong relationship between the *E* and *S* parameters was evident (Figure 4B,C). Particularly, the value of *S* becomes highly constrained over long timescales, hinting that most ARGs that have made it all the way to human pathogens should be close to neutral with respect to impact on host fitness. It is also interesting to note the relative increase in the importance of environmental processes over time (Figure 4A), which also relates to the impact of selection over time and how the environment presents a vastly larger number of bacteria with potentially fitness-neutral ARGs to recruit from.

Building on the assumption of neutral impact on host fitness (or, more precisely, neutral average impact on fitness over the run time of the model), we also ran the model with *S* fixed to 1 (i.e., no effects on fitness for any ARG). This is an interesting scenario as it caps *E* to the range between 10^–24^ and 10^–11^ (Figure S6), meaning that if fitness cost is neutral, currently observed ARGs in pathogens would have been present in somewhere between 10^6^ and 10^19^ bacterial cells at the start of the antibiotic era. However, other than that fixing *S* to 1 changes how the different processes depend on the *E* parameter (Figure S7), the model output is not substantially altered compared to the model with a variable *S* parameter.

We also compared the pre-existing model to the emergence model, in which ARGs are assumed not to have been present in bacteria at the start of the antibiotic era but had to develop their resistance function, i.e. emerge as ARGs, within that time frame. However, the results differed only slightly from the results of the pre-existing model (Figure S8). Based on the model results, the conceptual difference between the pre-existing model and emergence model is not sufficient to change the dependencies between the rest of the processes.

### Model Predictions

While data to infer the values of *D*, *S*, and *MH* are currently scarce, which is one of the major limitations of the model, it makes some predictions that may be testable within the foreseeable future. The respective results will substantially contribute to determine the crucial processes in antibiotic resistance development and thus help in the identification of effective intervention strategies:

(1) Fitness costs of ARGs: The model predicts that the majority of ARGs present in pathogens today should have very limited effects on fitness. The model caps the average fitness impact for ARGs currently present in human pathogens between −0.2 and +0.1% per generation, and 30 years into the future, the model predicts that the range of possible fitness effects will have narrowed even further (−0.15 to +0.1%; data not shown). By determining the fitness effects of carrying individual ARGs in their current hosts, considering inter- as well as intraspecies variability, the prediction that most ARGs impose a very minor fitness impact could be experimentally tested, although it would have to be validated across a large number of ARGs.
(2) The origin of ARGs: The model predicts that the most likely location of ARGs 70 years ago would have been in human-associated bacteria (Table 3). By tracking ARGs currently present in human pathogens across large datasets of bacterial genomes, it may be possible to trace the evolutionary history of these genes and thereby identify their likely hosts at the beginning of the antibiotic era. Such an attempt was recently made, corroborating the findings of our model^46^ and lending support to the idea that most ARGs may not originate in the environment. However, this analysis is complicated by the biased sampling of fully sequenced bacterial genomes, most of which originate from human specimens (https://gold.jgi.doe.gov/distribution). Thus, it could be expected that when performed today, such analysis would only be able to trace origins for ARGs that were present in human-associated bacteria 70 years ago. Notably, our model estimates that such genes would be more likely than not to make up the majority of all ARGs currently present in pathogens. Importantly, the rapid increase in sequencing capacity may make full-scale analysis of ARG origins using genomic data possible in the relatively near future, which would enable further testing of this prediction of the model.
(3) More data will enable additional specific predictions: Given that the origins of ARGs currently circulating in pathogens can be established, the range of possible predicted values of *D* narrows dramatically. If it can be shown that the proportion of ARGs in pathogens today that were already present in human-associated bacteria 70 years ago is not even close to 60%, the dispersal parameter *D* has to be above 10^–12^ (Table 3). Conversely, if *M* and *H* could be better determined by experiments, the model would predict the likely origins more precisely (Table S3). Just establishing a ball-park range for the mobilization rate (*M*) would dramatically restrict the possible outcomes of the model, particularly if *M* turns out to be at the extreme ends of the spectrum. Thus, a more precise determination of any of these parameters would enable several more specific predictions by the model.

### Early ARG Prevalence and Average Fitness Cost Are Linked

The model predicts a vast range of possible outcomes and parameter values; however, it serves its purpose to highlight which processes and parameters are interdependent and which ones are key to get a better understanding of how to mitigate antibiotic resistance development. A key aspect shaping the model is the strong relation between longevity of ARGs (largely determined by their fitness cost) and how widespread ARGs were across bacteria at the start of the antibiotic era (which likely serves as a proxy for how large the total genetic reservoir to recruit ARGs from still is today^47^). As the average fitness cost of carrying an ARG increases in the model (resulting in lower values of the *S* parameter), the number of available ARGs, either in the human microbiome, the environment, or both, needs to get bigger to compensate for that. As the model scenario plays out over a vast amount of time (in microbial terms), the possible range of average fitness costs or gains associated with ARG carriage becomes very small. Furthermore, the fitness impact of an ARG is highly context-dependent; a given ARG may be beneficial in some contexts but incur a fitness cost in others. Thus, the model essentially predicts that the ARGs present in pathogens today should have no or little fitness cost on average, which is in accordance with theoretical arguments we have put forth previously.^9,12^ As mentioned earlier, this prediction is, in principle, testable by large-scale experiments on the impacts of ARG carriage on bacterial fitness. Notably, negligible average fitness costs for ARGs would be associated with greater likelihood that they have been present in human pathogens all along. However, as most pathogens evidently do not carry a vast number of ARGs, it is likely that the average ARG is associated with a small, but significant, fitness cost. Overall, it is clear that the fitness cost of ARGs is a key property for their ability to make their way to and persist in human pathogens. The persistence is likely to be facilitated by exposure to low levels of antibiotics but also by mutational processes that lead to domestication of ARGs and MGEs, as well as other forms of fitness cost reductions, some of which may yet be unknown. In this context, a variety of mechanisms to reduce the fitness cost of ARGs are known but quantitative information on their occurrence and effect is still largely lacking. More research is needed to quantitatively determine the impact of domestication on selection of ARGs, as well as what mechanisms are available to bacteria for reducing fitness costs of ARGs. Furthermore, minimal selective concentrations must be determined for a much larger set of antibiotics, environmental conditions, and bacterial species, alone as well as in microbial communities, to provide actual scientific data in a field that currently relies largely on predictions of antibiotic effects on microbes.^48,49^

### Intervention Opportunities

Despite the large uncertainties associated with the model results, some useful intervention priorities can still be derived from this work (Table 4). First, due to the strong influence of selection and ARG fitness costs on the model outcomes, it is clear that there is a need for a deeper understanding of the settings that drive antibiotic resistance selection. Identifying threshold concentrations of antibiotics (and other selective agents, such as biocides^32,50^) that select or co-select for antibiotic resistance is crucial for determining which emission limits should be imposed on effluents from pharmaceutical production^51,52^ as well as from regular wastewater treatment.^53−55^ Currently, regulatory efforts are mostly based on predicted no-effect concentrations,^48,49^ and while this is a decent interim solution, solid experimental data on actual minimal selective concentrations in relevant settings are urgently needed.^10^

**Table 4:** Identified Knowledge Gaps and Research Needs that Must Be Addressed to Build Quantitative Risk Assessment Models and Better Mitigate ARG Recruitment

knowledge gap	research need	utility
settings that select for antibiotic resistance	minimal selective concentrations (MSCs) for more antibiotics, environmental conditions, and bacterial species, alone, in co-culture, and in complete communities	better sewage and wastewater treatment strategies, emission limits, and environmental quality standards for antibiotics
rate of bacterial dispersal between environmental compartments (and to humans)	quantitative observations of bacterial dispersal between environments, tracing spread of ARGs between environments over time, and better determination of human exposure to environmental bacteria	ability to discern if the environment has a significant role in the emergence of novel ARGs, improved quantitative risk assessment models for environmental antibiotic resistance, ability to limit spread of ARGs and resistant bacteria from the environment to humans, and improved wastewater treatment strategies
quantitative information on the occurrence and effect of mechanisms for fitness cost reduction and domestication of ARGs	quantitative understanding of genomic mechanisms and genes responsible for fitness cost reduction of ARGs	improved quantitative risk assessment models for environmental antibiotic resistance
rate of horizontal gene transfer between bacteria	measurement of transfer rates (especially for transformation and transduction) between a larger diversity of bacterial species and in a greater number of settings	improved quantitative risk assessment models for environmental antibiotic resistance and ability to curb transfer of ARGs between bacteria or reduce the number of settings where this can take place
rate of mobilization of genes from bacterial chromosomes to MGEs	development of proper experimental setups to measure mobilization and measurement of mobilization rates using such assays	improved quantitative risk assessment models for environmental antibiotic resistance and a better understanding of whether the environment acts as a source of ARGs to human pathogens, and thereby better prioritization of interventions

Finally, if we can gain a better understanding of how resistant bacteria and ARGs disseminate via the environment between compartments and, particularly, from the environment to humans, it would be possible to start designing mitigation strategies aiming to reduce the spread of resistant bacteria through the environmental route. Such interventions could be made either on the level of releases into the environment or an attempt to create barriers between humans and particular risk settings.^8^ Which of those would be most beneficial is, to a large extent, determined by the rate of dispersal of resistant bacteria from the environment to humans. Sadly, our current knowledge of the environmental dissemination routes is poor, mostly anecdotal and generally without quantitative measurements, which is also reflected in the simplistic definition of the dispersal parameter in our model.

### Outlook

In this paper, we present a model to describe the emergence of ARGs in human pathogens and populate it using the limited literature data that exist. We have intentionally kept the model and its parameters general in order to provide an overview of the respective importance of the individual processes that influence the appearance of new ARGs in human pathogens. However, the model still emphasizes important and interrelated processes that need more research attention in order to build more quantitatively accurate models. Importantly, the model constitutes an initial effort to create a conceptual framework for ARG emergence in pathogens,^9,12^ which is quantitative, and to highlight where more data is needed in order to make more precise predictions. As such, we hope that this modeling effort can spur more research, both into determining the model parameter ranges more precisely but also into more sophisticated modeling approaches that may better reveal the details on ARG emergence.

## Supporting Information Available

Detailed methods description, (Table S1) literature survey: mobilization, (Table S2) literature survey: conjugation, (Table S3), dependency of the modeled processes on the *MH* and *H* parameters, (Figure S1) relations between *E* and other parameters for the pre-existing model, (Figure S2) dependency of the different processes on the *H* parameter for the pre-existing model, (Figure S3), dependency of the different processes on the *MH* parameter for the pre-existing model, (Figure S4), dependency of the different processes on the *D* parameter for the pre-existing model, (Figure S5), dependency of the different processes on the *MH* parameter divided by the *H* parameter (resulting in an estimated *M* parameter) for the pre-existing model, (Figure S6), valid parameter ranges and process rates for the pre-existing model with *S* being fixed to 1, (Figure S7), differences between how processes depend on *E* for the pre-existing model and the model where *S* is fixed to 1, (Figure S8), valid parameter ranges and process rates for the emergence model, links to the code of the two models and full results, and references (PDF)

## References

[ref1] O’Neill J. Tackling Drug-resistant Infections Globally: Final Report and Recommendations–The Review on Antimicrobial Resistance; Wellcome Trust and HM Government, 2016.

[ref2] European Commission. A European One Health Action Plan against Antimicrobial Resistance (AMR); European Commission, 2017.

[ref3] Ashbolt N. J.; Amézquita A.; Backhaus T.; Borriello P.; Brandt K. K.; Collignon P.; Coors A.; Finley R.; Gaze W. H.; Heberer T.; Lawrence J. R.; Larsson D. G. J.; McEwen S. A.; Ryan J. J.; Schönfeld J.; Silley P.; Snape J. R.; Van den Eede C.; Topp E. Human Health Risk Assessment (HHRA) for Environmental Development and Transfer of Antibiotic Resistance. Environ. Health Perspect. 2013, 121, 993–1001. 10.1289/ehp.1206316. 23838256 PMC3764079

[ref4] Finley R. L.; Collignon P.; Larsson D. G. J.; McEwen S. A.; Li X.-Z.; Gaze W. H.; Reid-Smith R.; Timinouni M.; Graham D. W.; Topp E. The Scourge of Antibiotic Resistance: The Important Role of the Environment. Clin. Infect. Dis. 2013, 57, 704–710. 10.1093/cid/cit355. 23723195

[ref5] Gaze W. H.; Krone S. M.; Larsson D. G. J.; Li X.-Z.; Robinson J. A.; Simonet P.; Smalla K.; Timinouni M.; Topp E.; Wellington E. M.; Wright G. D.; Zhu Y.-G. Influence of Humans on Evolution and Mobilization of Environmental Antibiotic Resistome. Emerging Infect. Dis. 2013, 19, e120871 10.3201/eid1907.120871. PMC3713965 23764294

[ref6] Pruden A.; Larsson D. G. J.; Amézquita A.; Collignon P.; Brandt K. K.; Graham D. W.; Lazorchak J. M.; Suzuki S.; Silley P.; Snape J. R.; Topp E.; Zhang T.; Zhu Y.-G. Management Options for Reducing the Release of Antibiotics and Antibiotic Resistance Genes to the Environment. Environ. Health Perspect. 2013, 121, 878–885. 10.1289/ehp.1206446. 23735422 PMC3734499

[ref7] Berendonk T. U.; Manaia C. M.; Merlin C.; Fatta-Kassinos D.; Cytryn E.; Walsh F.; Bürgmann H.; Sørum H.; Norström M.; Pons M.-N.; Kreuzinger N.; Huovinen P.; Stefani S.; Schwartz T.; Kisand V.; Baquero F.; Martinez J. L. Tackling Antibiotic Resistance: The Environmental Framework. Nat. Rev. Microbiol. 2015, 13, 310–317. 10.1038/nrmicro3439. 25817583

[ref8] Bengtsson-Palme J.; Hess S. Strategies to Reduce or Eliminate Resistant Pathogens in the Environment. In Antimicrobial Drug Resistance; Wiley, 2019; 637–673.

[ref9] Bengtsson-Palme J.; Kristiansson E.; Larsson D. G. J. Environmental Factors Influencing the Development and Spread of Antibiotic Resistance. FEMS Microbiol. Rev. 2018, 42, 25 10.1093/femsre/fux053. PMC5812547 29069382

[ref10] Larsson D. G. J.; Andremont A.; Bengtsson-Palme J.; Brandt K. K.; de Roda Husman A. M.; Fagerstedt P.; Fick J.; Flach C.-F.; Gaze W. H.; Kuroda M.; Kvint K.; Laxminarayan R.; Manaia C. M.; Nielsen K. M.; Plant L.; Ploy M.-C.; Segovia C.; Simonet P.; Smalla K.; Snape J.; Topp E.; van Hengel A. J.; Verner-Jeffreys D. W.; Virta M. P. J.; Wellington E. M.; Wernersson A.-S. Critical Knowledge Gaps and Research Needs Related to the Environmental Dimensions of Antibiotic Resistance. Environ. Int. 2018, 117, 132–138. 10.1016/j.envint.2018.04.041. 29747082

[ref11] Martinez J. L.; Coque T. M.; Baquero F. What is a resistance gene? Ranking risk in resistomes. Nat. Rev. Microbiol. 2015, 13, 116–123. 10.1038/nrmicro3399. 25534811

[ref12] Bengtsson-Palme J. Assessment and Management of Risks Associated With Antibiotic Resistance in the Environment. In Management of Emerging Public Health Issues and Risks; Elsevier, 2019; pp. 243–263.

[ref13] Knight G. M.; Davies N. G.; Colijn C.; Coll F.; Donker T.; Gifford D. R.; Glover R. E.; Jit M.; Klemm E.; Lehtinen S.; Lindsay J. A.; Lipsitch M.; Llewelyn M. J.; Mateus A. L. P.; Robotham J. V.; Sharland M.; Stekel D.; Yakob L.; Atkins K. E. Mathematical modelling for antibiotic resistance control policy: do we know enough?. BMC Infect. Dis. 2019, 19, 1011 10.1186/s12879-019-4630-y. 31783803 PMC6884858

[ref14] Baker M.; Hobman J. L.; Dodd C. E. R.; Ramsden S. J.; Stekel D. J. Mathematical modelling of antimicrobial resistance in agricultural waste highlights importance of gene transfer rate. FEMS Microbiol. Ecol. 2016, 92, fiw040 10.1093/femsec/fiw040. 26906100

[ref15] Blanquart F.; Lehtinen S.; Fraser C. An evolutionary model to predict the frequency of antibiotic resistance under seasonal antibiotic use, and an application to Streptococcus pneumoniae. Proc. R. Soc. B 2017, 284, 20170679 10.1098/rspb.2017.0679. PMC5454275 28566489

[ref16] Blanquart F.; Lehtinen S.; Lipsitch M.; Fraser C. The evolution of antibiotic resistance in a structured host population. J. R. Soc., Interface 2018, 15, 20180040 10.1098/rsif.2018.0040. 29925579 PMC6030642

[ref17] D’Costa V. M.; King C. E.; Kalan L.; Morar M.; Sung W. W. L.; Schwarz C.; Froese D.; Zazula G.; Calmels F.; Debruyne R.; Golding G. B.; Poinar H. N.; Wright G. D. Antibiotic Resistance Is Ancient. Nature 2011, 477, 457–461. 10.1038/nature10388. 21881561

[ref18] Bhullar K.; Waglechner N.; Pawlowski A.; Koteva K.; Banks E. D.; Johnston M. D.; Barton H. A.; Wright G. D. Antibiotic resistance is prevalent in an isolated cave microbiome. PLoS One 2012, 7, e34953 10.1371/journal.pone.0034953. 22509370 PMC3324550

[ref19] Berglund F.; Österlund T.; Boulund F.; Marathe N. P.; Larsson D. G. J.; Kristiansson E. Identification and reconstruction of novel antibiotic resistance genes from metagenomes. Microbiome 2019, 7, 52 10.1186/s40168-019-0670-1. 30935407 PMC6444489

[ref20] Crippen C. S.; Rothrock M. J. Jr.; Sanchez S.; Szymanski C. M. Multidrug Resistant Acinetobacter Isolates Release Resistance Determinants Through Contact-Dependent Killing and Bacteriophage Lysis. Front. Microbiol. 2020, 11, 1918 10.3389/fmicb.2020.01918. 32922376 PMC7456956

[ref21] Franke A. E.; Clewell D. B. Evidence for a Chromosome-Borne Resistance Transposon (Tn916) in Streptococcus Faecalis That Is Capable of “Conjugal” Transfer in the Absence of a Conjugative Plasmid. J. Bacteriol. 1981, 145, 494–502. 10.1128/JB.145.1.494-502.1981. 6257641 PMC217299

[ref22] Scott J. R.; Kirchman P. A.; Caparon M. G. An Intermediate in Transposition of the Conjugative Transposon Tn916. Proc. Natl. Acad. Sci. U. S. A. 1988, 85, 4809–4813. 10.1073/pnas.85.13.4809. 2838847 PMC280525

[ref23] Torres O. R.; Korman R. Z.; Zahler S. A.; Dunny G. M. The Conjugative Transposon Tn925: Enhancement of Conjugal Transfer by Tetracycline in Enterococcus Faecalis and Mobilization of Chromosomal Genes in Bacillus Subtilis and E. Faecalis. Mol. Gen. Genet. 1991, 225, 395–400. 10.1007/BF00261679. 1850085

[ref24] Poyart C.; Celli J.; Trieu-Cuot P. Conjugative Transposition of Tn916-Related Elements from Enterococcus Faecalis to Escherichia Coli and Pseudomonas Fluorescens. Antimicrob. Agents Chemother. 1995, 39, 500–506. 10.1128/AAC.39.2.500. 7726521 PMC162567

[ref25] Manson J. M.; Hancock L. E.; Gilmore M. S. Mechanism of Chromosomal Transfer of Enterococcus Faecalis Pathogenicity Island, Capsule, Antimicrobial Resistance, and Other Traits. Proc. Natl. Acad. Sci. U. S. A. 2010, 107, 12269–12274. 10.1073/pnas.1000139107. 20566881 PMC2901427

[ref26] Lang J. M.; Eisen J. A.; Zivkovic A. M. The Microbes We Eat: Abundance and Taxonomy of Microbes Consumed in a Day’s Worth of Meals for Three Diet Types. PeerJ 2014, 2, e659 10.7717/peerj.659. 25538865 PMC4266855

[ref27] Leonard A. F. C.; Zhang L.; Balfour A. J.; Garside R.; Gaze W. H. Human Recreational Exposure to Antibiotic Resistant Bacteria in Coastal Bathing Waters. Environ. Int. 2015, 82, 92–100. 10.1016/j.envint.2015.02.013. 25832996

[ref28] Leonard A. F. C.; Zhang L.; Balfour A. J.; Garside R.; Hawkey P. M.; Murray A. K.; Ukoumunne O. C.; Gaze W. H. Exposure to and Colonisation by Antibiotic-Resistant E. Coli in UK Coastal Water Users: Environmental Surveillance, Exposure Assessment, and Epidemiological Study (Beach Bum Survey). Environ. Int. 2018, 114, 326–333. 10.1016/j.envint.2017.11.003. 29343413

[ref29] Sender R.; Fuchs S.; Milo R. Revised Estimates for the Number of Human and Bacteria Cells in the Body. 2016, 14 ( (8), ), e1002533, 10.1371/journal.pbio.1002533. PMC4991899 27541692

[ref30] Kallmeyer J.; Pockalny R.; Adhikari R. R.; Smith D. C.; D’Hondt S. Global Distribution of Microbial Abundance and Biomass in Subseafloor Sediment. Proc. Natl. Acad. Sci. U. S. A. 2012, 109, 16213–16216. 10.1073/pnas.1203849109. 22927371 PMC3479597

[ref31] Gregory T. R. Synergy between Sequence and Size in Large-Scale Genomics. Nat. Rev. Genet. 2005, 6, 699–708. 10.1038/nrg1674. 16151375

[ref32] Pal C.; Bengtsson-Palme J.; Kristiansson E.; Larsson D. G. J. Co-Occurrence of Resistance Genes to Antibiotics, Biocides and Metals Reveals Novel Insights into Their Co-Selection Potential. BMC Genomics 2015, 16, 964 10.1186/s12864-015-2153-5. 26576951 PMC4650350

[ref33] Martinez J. L. Antibiotics and Antibiotic Resistance Genes in Natural Environments. Science 2008, 321, 365–367. 10.1126/science.1159483. 18635792

[ref34] Allen H. K.; Donato J.; Wang H. H.; Cloud-Hansen K. A.; Davies J.; Handelsman J. Call of the Wild: Antibiotic Resistance Genes in Natural Environments. Nat. Rev. Microbiol. 2010, 8, 251–259. 10.1038/nrmicro2312. 20190823

[ref35] Baumgardner D. J. Soil-Related Bacterial and Fungal Infections. J. Am. Board. Fam. Med. 2012, 25, 734–744. 10.3122/jabfm.2012.05.110226. 22956709

[ref36] Argudín M. A.; Deplano A.; Meghraoui A.; Dodémont M.; Heinrichs A.; Denis O.; Nonhoff C.; Roisin S. Bacteria from Animals as a Pool of Antimicrobial Resistance Genes. Antibiotics 2017, 6, 12 10.3390/antibiotics6020012. PMC5485445 28587316

[ref37] Iramiot J. S.; Kajumbula H.; Bazira J.; Kansiime C.; Asiimwe B. B. Antimicrobial Resistance at the Human-Animal Interface in the Pastoralist Communities of Kasese District, South Western Uganda. Sci. Rep. 2020, 10, 14737 10.1038/s41598-020-70517-w. 32895433 PMC7477235

[ref38] Pruden A.; Alcalde R. E.; Alvarez P. J. J.; Ashbolt N.; Bischel H.; Capiro N. L.; Crossette E.; Frigon D.; Grimes K.; Haas C. N.; Ikuma K.; Kappell A.; LaPara T.; Kimbell L.; Li M.; Li X.; McNamara P.; Seo Y.; Sobsey M. D.; Sozzi E.; Navab-Daneshmand T.; Raskin L.; Riquelme M. V.; Vikesland P.; Wigginton K.; Zhou Z. An Environmental Science and Engineering Framework for Combating Antimicrobial Resistance. Environ. Eng. Sci. 2018, 35, 1005–1011. 10.1089/ees.2017.0520.

[ref39] Sun D.; Zhang Y.; Mei Y.; Jiang H.; Xie Z.; Liu H.; Chen X.; Shen P. Escherichia Coli Is Naturally Transformable in a Novel Transformation System. FEMS Microbiol. Lett. 2006, 265, 249–255. 10.1111/j.1574-6968.2006.00503.x. 17069625

[ref40] Etchuuya R.; Ito M.; Kitano S.; Shigi F.; Sobue R.; Maeda S. Cell-to-Cell Transformation in Escherichia Coli: A Novel Type of Natural Transformation Involving Cell-Derived DNA and a Putative Promoting Pheromone. PLoS One 2011, 6, e16355 10.1371/journal.pone.0016355. 21283723 PMC3024429

[ref41] Jutkina J.; Rutgersson C.; Flach C.-F.; Larsson D. G. J. An Assay for Determining Minimal Concentrations of Antibiotics That Drive Horizontal Transfer of Resistance. Sci. Total Environ. 2016, 548, 131–138. 10.1016/j.scitotenv.2016.01.044. 26802341

[ref42] Kneis D.; Berendonk T. U.; Heß S. High Prevalence of Colistin Resistance Genes in German Municipal Wastewater. Sci. Total Environ. 2019, 694, 133454 10.1016/j.scitotenv.2019.07.260. 31398645

[ref43] Balcazar J. L. Bacteriophages as Vehicles for Antibiotic Resistance Genes in the Environment. PLoS Pathog. 2014, 10, e1004219 10.1371/journal.ppat.1004219. 25078987 PMC4117541

[ref44] Ross J.; Topp E. Abundance of Antibiotic Resistance Genes in Bacteriophage Following Soil Fertilization With Dairy Manure or Municipal Biosolids, and Evidence for Potential Transduction. Appl. Environ. Microbiol. 2015, 7905 10.1128/AEM.02363-15. 26341211 PMC4616940

[ref45] Dong P.; Wang H.; Fang T.; Wang Y.; Ye Q. Assessment of Extracellular Antibiotic Resistance Genes (EARGs) in Typical Environmental Samples and the Transforming Ability of EARG. Environ. Int. 2019, 125, 90–96. 10.1016/j.envint.2019.01.050. 30711653

[ref46] Ebmeyer S.; Kristiansson E.; Larsson D. G. J. A Framework for Identifying the Recent Origins of Mobile Antibiotic Resistance Genes. Commun. Biol. 2021, 4, 8 10.1038/s42003-020-01545-5. 33398069 PMC7782503

[ref47] Bengtsson-Palme J.; Larsson D. G. J. Antibiotic Resistance Genes in the Environment: Prioritizing Risks. Nat. Rev. Microbiol. 2015, 13, 396 10.1038/nrmicro3399-c1. 25915637

[ref48] Bengtsson-Palme J.; Larsson D. G. J. Concentrations of Antibiotics Predicted to Select for Resistant Bacteria: Proposed Limits for Environmental Regulation. Environ. Int. 2016, 86, 140–149. 10.1016/j.envint.2015.10.015. 26590482

[ref49] Tell J.; Caldwell D. J.; Häner A.; Hellstern J.; Hoeger B.; Journel R.; Mastrocco F.; Ryan J. J.; Snape J.; Straub J. O.; Vestel J. Science-Based Targets for Antibiotics in Receiving Waters from Pharmaceutical Manufacturing Operations. Integr. Environ. Assess. Manage. 2019, 15, 312–319. 10.1002/ieam.4141. PMC6849714 30884149

[ref50] Wales A.; Davies R. Co-Selection of Resistance to Antibiotics, Biocides and Heavy Metals, and Its Relevance to Foodborne Pathogens. Antibiotics 2015, 4, 567–604. 10.3390/antibiotics4040567. 27025641 PMC4790313

[ref51] Larsson D. G. J.; de Pedro C.; Paxeus N. Effluent from Drug Manufactures Contains Extremely High Levels of Pharmaceuticals. J. Hazard. Mater. 2007, 148, 751–755. 10.1016/j.jhazmat.2007.07.008. 17706342

[ref52] Bengtsson-Palme J.; Gunnarsson L.; Larsson D. G. J. Can Branding and Price of Pharmaceuticals Guide Informed Choices towards Improved Pollution Control during Manufacturing?. J. Cleaner Prod. 2018, 171, 137–146. 10.1016/j.jclepro.2017.09.247.

[ref53] Giger W.; Alder A. C.; Golet E.; Kohler H.; McArdell C.; Molnar E.; Siegrist H.; Suter M. Occurrence and Fate of Antibiotics as Trace Contaminants in Wastewaters, Sewage Sludges, and Surface Waters. CHIMIA Int. J. Chem 2003, 57, 485–491. 10.2533/000942903777679064.

[ref54] Lindberg R. H.; Björklund K.; Rendahl P.; Johansson M. I.; Tysklind M.; Andersson B. A. V. Environmental Risk Assessment of Antibiotics in the Swedish Environment with Emphasis on Sewage Treatment Plants. Water Res. 2007, 41, 613–619. 10.1016/j.watres.2006.11.014. 17187841

[ref55] Xu J.; Xu Y.; Wang H.; Guo C.; Qiu H.; He Y.; Zhang Y.; Li X.; Meng W. Occurrence of Antibiotics and Antibiotic Resistance Genes in a Sewage Treatment Plant and Its Effluent-Receiving River. Chemosphere 2015, 119, 1379–1385. 10.1016/j.chemosphere.2014.02.040. 24630248

