# Supplementary material for: What Is the Role of the Environment in the Emergence of Novel Antibiotic Resistance Genes? A Modeling Approach
Source: Environ Sci Technol. 2021 Nov 18;55(23):15734–43. doi: 10.1021/acs.est.1c02977 (PMC8655980; doi:10.1021/acs.est.1c02977)
Supplement: Supplementary file 1 — es1c02977_si_001.pdf [file es1c02977_si_001.pdf]

What is the role of the environment in the emergence of novel antibiotic resistance genes? – A modelling approach

Johan Bengtsson-Palme[1,2*], Viktor Jonsson[3], Stefanie Heß[4]

[1] Department of Infectious Diseases, Institute of Biomedicine, The Sahlgrenska Academy, University of Gothenburg, Guldhedsgatan 10, SE-413 46, Gothenburg, Sweden
[2] Centre for Antibiotic Resistance research (CARe) at University of Gothenburg, Gothenburg, Sweden
[3] Integrated Science Lab, Department of Physics, Umeå University, SE-901 87 Umeå, Sweden
[4] Institute of Microbiology, Technische Universität Dresden, Zellescher Weg 20b, 01847 Dresden, Germany

* Corresponding author: johan.bengtsson-palme@microbiology.se

Supplementary Information - Table of content

Detailed methods description	S2
Table S1: Literature survey: Mobilzation	S5
Table S2: Literature survey: Conjugation	S6
Table S3: Dependency of the modelled processes on the MH and H parameters	S7
Figure S1: Relations between E and other parameters for the pre-existing model	S8
Figure S2: Dependency of the different processes on the H parameter for the pre-existing model	S9
Figure S3: Dependency of the different processes on the MH parameter for the pre-existing model	S10
Figure S4: Dependency of the different processes on the D parameter for the pre-existing model	S11
Figure S5: Dependency of the different processes on the MH parameter divided by the H parameter (resulting in an estimated M parameter) for the pre-existing model after 70 years of simulated time	S12
Figure S6: Valid parameter ranges and process rates for the pre-existing model with S being fixed to 1	S13
Figure S7: Differences between how processes depend on E for the pre-existing model and the model where S is fixed to 1	S14
Figure S8: Valid parameter ranges and process rates for the emergence model	S15
Links to the code of the two models and full results	S16
References (supplement)	S17

Detailed methods description

Implementation of the model

We consider two different scenarios in our model. In the first scenario, ARGs that later end up in human pathogens already pre-existed in bacteria in some setting at the start of the antibiotic era (around 70 years ago), and only needed to be, in some way, transferred to human pathogens (Main text, Table 1). In the second model scenario, we assume that ARGs did not have a resistance function at the start of the modelled time period but needed to first emerge as resistance factors. We will refer to the latter scenario as the "emergence model" and the first scenario as the "pre-existing model".

The endpoint of the proposed models is a mobilized ARG observed in a human pathogen. Several different chains of events may lead from the first appearance of an ARG to its occurrence in a pathogenic strain (Main text, Fig. 1). These combinations of events are summarized in the model as six different pathways. First, an ARG may have existed directly on a mobile genetic element in a human pathogen living in a human being (E1). Secondly, an ARG could have already existed on the chromosome of a human commensal bacteria. After mobilization, the ARG could then be horizontally transferred to a human pathogen (E2). Thirdly, an ARG could originate from a mobile genetic element in a commensal species belonging to the human microbiome. In this scenario, it would then be directly transferrable into a human pathogen via HGT (E3). These first three processes all target pre-existence of ARGs in bacteria directly associated with humans. However, it is also plausible that ARGs may have originated in bacteria living in the environment and subsequently been disseminated to humans. The simplest case would be an ARG that originated from a human pathogen present in the environment. It could then be disseminated between the compartments before ending up in humans (E4). Alternatively, a chromosomally encoded ARG could have existed among environmental bacteria that are not pathogenic. Subsequently, that ARG needs to be mobilized onto a plasmid or as a transposon, spread via horizontal gene transfer (HGT, referred to with the letter 'H' in the model) and be transferred to human pathogens. Humans might pick up the bacterium harbouring the ARG, for instance, with food or while swimming (E5). An ARG could also have pre-existed on a mobile genetic element in an environmental bacterium. Via transformation, transduction or conjugation (H), it could then get transferred to a human pathogen, which can subsequently be taken up by humans (E6).

Time plays an important role in all processes relating to the spread and maintenance of ARGs. For mobilization (M), horizontal gene transfer (H) and the dissemination processes (D), it is important that the microbe carrying the gene survives. Many human pathogenic species are known to have a rather short lifetime outside of their host [1,2]. Furthermore, the fitness effect of ARG carriage on population size over time is cumulative, affecting the population expansion rate (S) exponentially, in contrast to the other model parameters. The dissemination efficiency is additionally influenced by the distance to humans in terms of space and time. Human exposure is a critical factor for the dissemination as well as the appearance of the ARG in pathogens in the human microbiome. Humans are exposed to bacteria in many different ways through which uptake via food seems to be the most important one in terms of bacterial exposure per day [3].

Each of the six pathways shown in Fig. 1 were expressed as equations (Main text, Table 1) with parameters defined in Table 2 (in the main text). The interpretation of each equation is the number of mobilized ARGs in pathogens at time t contributed by the corresponding pathway. Both models (the main "pre-existing" model and the "emergence" model) were implemented in R as follows, only differing in the meaning and range of the parameter E (Main text, Table 2). Each parameter was randomly selected from its log10 transformed range (see Main text, Table 2) under a uniform distribution. For each set of randomly selected parameters, the result of the equations for the different considered processes were calculated (Main text, Table 1). Next, the total appearance was calculated based on these sub-equations according to this formula:

$$App(t) = E_1(t)+E_2(t)+E_3(t)+E_4(t)+E_5(t)+E_6(t)$$

where E_1 to E_6 each represent the contribution of ARGs by a particular process, expressed as total contributed ARGs over the entire model timeframe, and t is the time in days, resulting in the total appearance (App) representing the total observed ARGs at time t. The true total appearance was approximated from the ResFinder database [4], which depending on how gene variants are counted held on the order of 700 to 2200 unique ARGs in September 2019. By assuming that these genes would all have appeared in the past 70 years since antibiotics were widely introduced, that gives us an expected appearance rate of around 9 to 29 ARGs per year, or 0.025 to 0.079 per day (Main text, Table 2). If the modelled total appearance fell within the expected interval, the model parameters were saved. This procedure was iterated until 10,000 valid sets of model parameters had been obtained for each modelled time point and set of parameter ranges.

Defining the probability boundaries for antibiotic resistance development

In order to populate the model, measured values for the individual parameters were taken from literature (Supplementary Tables S1 and S2), and the upper and lower boundaries were fed into the model (Supplementary Table S1). For the rates of horizontal gene transfer (H) most studies were found for the conjugative transfer of genes. Data for both intra- and interspecies transfer of various naturally occurring and artificially constructed plasmids are available. These have been included as a first approximation in the model, bearing in mind that this is laboratory data and that their transferability to the respective ecosystems has been little studied so far. At this time, we have limited knowledge concerning the role of transduction and transformation for the distribution of resistance genes in the respective ecosystems, although recent studies have suggested that they may be important in the transfer of ARGs between bacteria [5]. Due to the lack of quantitative data, these two processes were not considered for the estimation of the parameter H.

Unfortunately, there are no direct measurements for the mobilization parameter (M) that have been obtained independent of HGT. Most studies have the following design in common [6–10]; the resistance encoded by the donor is not mobile and can only be detected in the recipient once it has been mobilized and transferred. Thus, we do not have a measure of the M parameter, and instead we have used the combined parameter MH (mobilization and horizontal gene transfer) where experimental data can be obtained from these studies.

To get an estimate for the dissemination of ARGs from the environment to humans (D), the number of eaten bacteria per day were used as a proxy [3]. In this study, the authors counted the number of colony forming units of meals for three different diet types. In this context, other transfer routes are also conceivable, e.g., the absorption of bacteria by swallowing water while bathing [11,12]. Such dissemination pathways can be of decisive importance when considering individual systems. However, they are not explicitly further considered in this model, which is intentionally kept general, as we aim to accommodate all possible dispersal scenarios in one single parameter.

Finally, some parameters were calculated based on the estimates of the number of humans on earth (7.8 billion; http://www.worldometers.info), the number of bacterial cells in/on the human body (3.8×10^{13}) [13] and the total number of bacterial cells on earth (on the order of 10^{30}) [14]. These values were used to define ranges for Pph, Ph, Pp and E (Main text, Table 2). We also estimate a typical bacterial genome to contain 1500 to 7500 genes [15], that around 7% of bacterial cells carry a conjugable plasmid, and that a typical plasmid carries 50-100 genes [16], which was used to constrain the Pm parameter. The S parameter represents the overall fitness impact of carrying an ARG and implicitly also involves the survival rate for a bacterium carrying an ARG, relative to a non-carrier. S is defined as the population expansion rate per day in the model, so when S is equal to one the average ARG would have no impact on the expansion of the population, i.e. the average ARG would overall be fitness neutral.

Limitations of the model

It is important to note that we have intentionally kept the model and its parameters general, in order to provide an overview of the respective importance of the individual processes that influence the appearance of new resistance genes in human pathogens. This is likely also one of the reasons why the model predictions are in many cases associated with very wide ranges. However, the model still emphasizes important and interrelated processes that need more research attention in order to build more quantitatively accurate models. Furthermore, by using specific rates for particular ecosystems as input for the model, we believe that our model could be used to assess the probabilities for the contribution of specific pathways for resistance development and dissemination.

A further limitation to the model is that the variable associated with the fitness cost of ARGs (S) represents a variety of different processes and parameters related to bacterial survival and proliferation. As we did not find an unambiguous way to tease these components apart, they are all captured by this single parameter, which of course results in an oversimplification of the real world. For these reasons, the role of S has to be interpreted with some caution. First of all, S represents the *average* fitness impact of the *average* ARG over the entire run time of the model (which in most of the data we have presented in this paper is 70 years). This means that S encapsulates scenarios where an ARG may some of the time have a positive fitness impact (such as during antibiotic selection) and at other times a slightly negative effect on fitness (e.g., in the absence of antibiotic selection). The final value of S therefore represents the expected overall fitness impact of ARG carriage, not the specific fitness of any single ARG currently encountered in pathogens. Furthermore, S also implicitly includes a constraint on the survival rate for a bacterium carrying an ARG. The way S is defined in the model, it is related to the population expansion rate per generation. In other words, if S is equal to one, the average ARG is expected to have no impact on bacterial growth and, thus, the population expansion (i.e. it is fitness neutral). However, given the relatively quick doubling rate of bacteria, a value only slightly deviating from one will rapidly result in the gene propagating through bacterial populations or quickly disappearing, which explains the tendency of S to approach one the longer the simulated time of the model.

Supplementary Table S1. Literature survey: Mobilization

Study	Species	Test system	Events/Day (average)	Remark
Torres et al., 1991 [8]	*Enterococcus faecalis*	Tn925	1.89E-07	Also includes a HGT step
Manson et al., 2010 [10]	*Enterococcus faecalis*	*E. faecalis* PAI	4.00×10^{-10}	Also includes a HGT step
Poyart et al., 1995 [9]	*Enterococcus faecalis*	Tn916	1.48×10^{-4}	Also includes a HGT step
Poyart et al., 1995 [9]	*Enterococcus faecalis*	Tn916	5.67×10^{-9}	Also includes a HGT step
Poyart et al., 1995 [9]	*Enterococcus faecalis*	Tn916	2.40×10^{-9}	Also includes a HGT step
Scott et al., 1988 [7]	*Bacillus subtilis*	Tn916	1.26×10^{-9}	Also includes a HGT step
Franke and Clewell, 1981 [6]	*Streptococcus faecalis*	Tn916	9.95×10^{-9}	Also includes a HGT step

Supplementary Table S2. Literature survey: Conjugation

Study	Species	Test system	Events/Day (average)	Remark
Hausner and Wuertz, 1999 [17]	*Escherichia coli*	pRK415	5.76×10^{-2}	
Wan et al., 2011 [18]	*Escherichia coli*	F-plasmid	2.16×10^{2}	
Simonsen et al., 1990 [19]	*Escherichia coli*	IncFII-plasmid R1	2.40×10^{-10}	
Andrup and Andersen, 1999 [20]	*Escherichia coli*	F-plasmid	2.16×10^{2}	
Andrup and Andersen, 1999 [20]	*Enterococcus sp.*	pCF10	4.18×10^{2}	
Zhong et al., 2012 [21]	*Escherichia coli*	pB10	3.12×10^{-2}	
Jutkina et al., 2016 [22]	*Escherichia coli*	MGEs derived from wastewater	3.84×10^{-5}	Stress: Sub-MIC tetracycline concentration
Normander et al., 1998 [23]	*Pseudomonas putida*	TOL-plasmid	4.46×10^{-3}	
Suhartono and Sarvin, 2016 [24]	*Escherichia coli*	Plasmids derived from river water	1.60×10^{-3}	Stress: 20 mg/L nalidixic acid and 80 mg/L sulfamethoxazole or 20 mg/L streptomycin and 16 mg/L tetracycline

Supplementary Table S3. Dependency of the modelled processes on the MH and H parameters

**Origin**	**MH:10^{-15} to 10^{-13}**	**MH:10^{-13} to 10^{-10}**	**MH:10^{-10} to 10^{-7}**	**MH:10^{-7} to 10^{-4}**	**MH:10^{-4} to 10^{-2}**
E1: pathogens in the human microbiome	26.7%	21.7%	0.588%	0.00194%	<0.00001%
E2: non-pathogenic human-associated bacteria	0.00659%	1.56%	36.3%	83.4%	96.0%
E3: MGEs in human-associated bacteria	70.1%	73.7%	59.4%	12.4%	1.02%
E4: pathogenic bacteria in the environment	<0.00001%	<0.00001%	<0.00001%	<0.00001%	<0.00001%
E5: chromosomes of environmental bacteria	0.00030%	0.0633%	1.38%	3.65%	2.92%
E6: MGEs in the environment	3.22%	2.98%	2.25%	0.545%	0.0311%
**Origin**	**H:10^{-11} to 10^{-9}**	**H:10^{-9} to 10^{-7}**	**H:10^{-7} to 10^{-5}**	**H:10^{-5} to 10^{-3}**	**H:10^{-3} to 10^{-1}**
E1: pathogens in the human microbiome	99.0%	73.3%	3.63%	0.0394%	0.00033%
E2: non-pathogenic human-associated bacteria	0.717%	5.90%	3.07%	0.303%	0.226%
E3: MGEs in human-associated bacteria	0.231%	19.7%	89.8%	95.6%	96.3%
E4: pathogenic bacteria in the environment	0.00001%	0.00001%	<0.00001%	<0.00001%	<0.00001%
E5: chromosomes of environmental bacteria	0.0326%	0.246%	0.115%	0.0130%	0.00087%
E6: MGEs in the environment	0.0105%	0.822%	3.36%	4.09%	3.72%

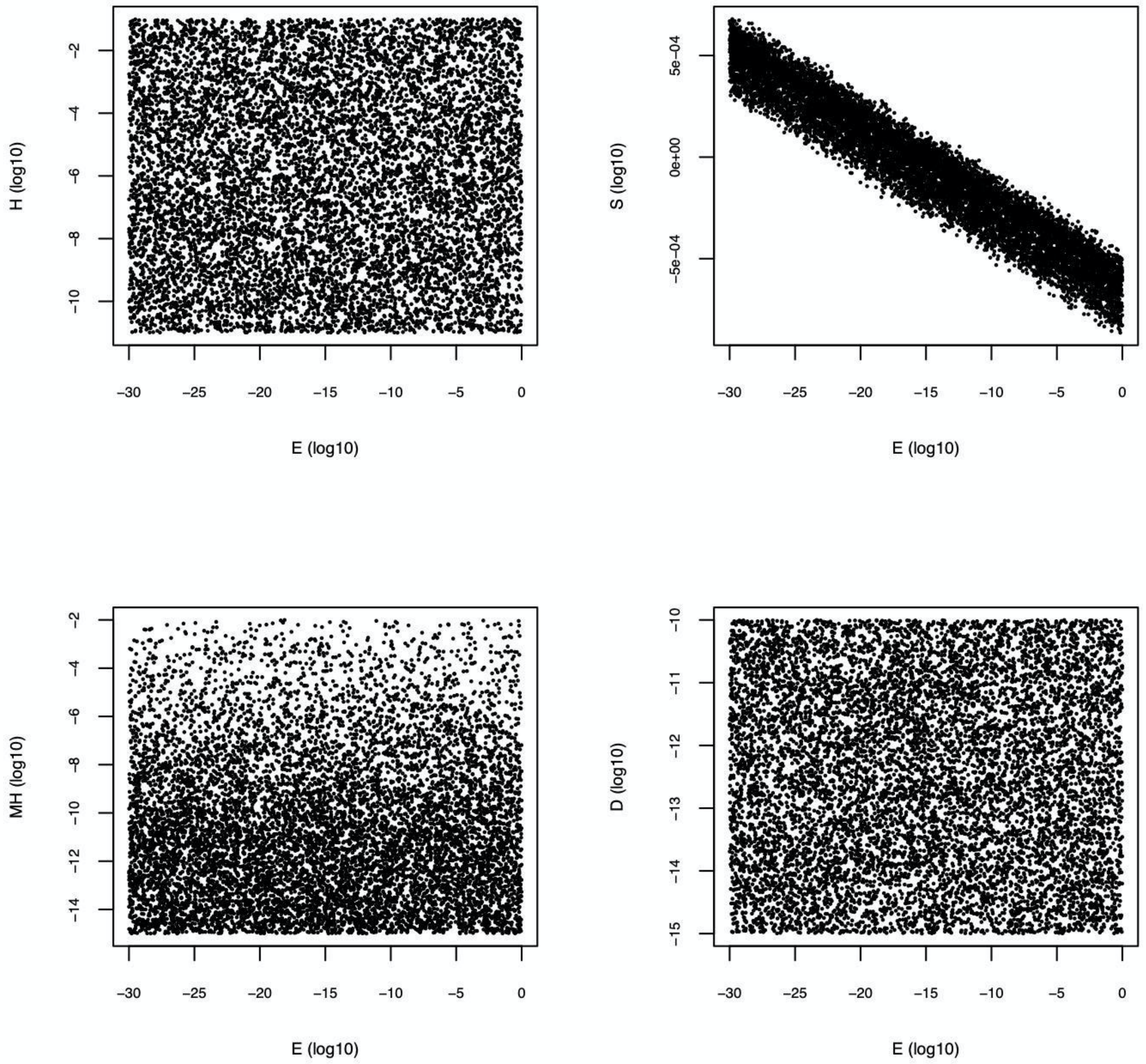

Supplementary Fig. S1. Relations between E and other parameters for the pre-existing model after 70 years of simulated time.

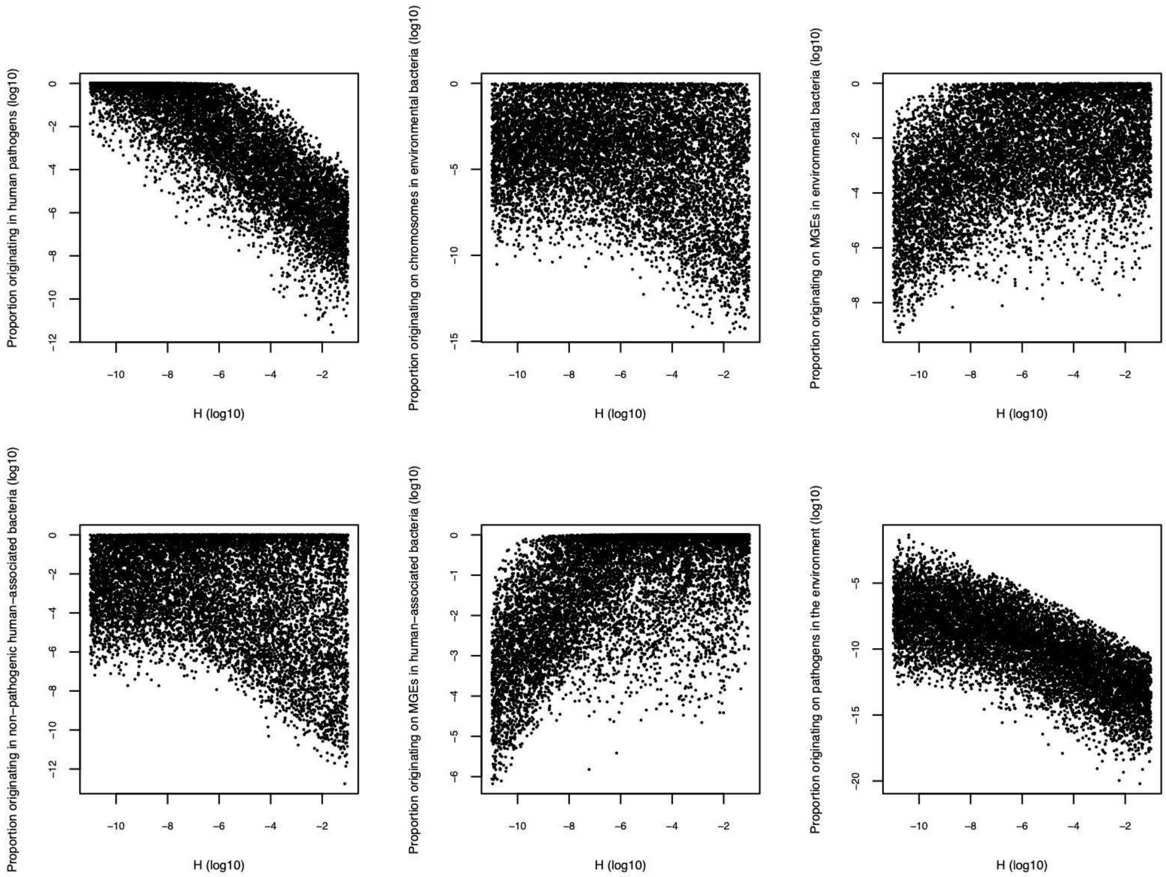

Supplementary Fig. S2. Dependency of the different processes on the H parameter for the pre-existing model after 70 years of simulated time.

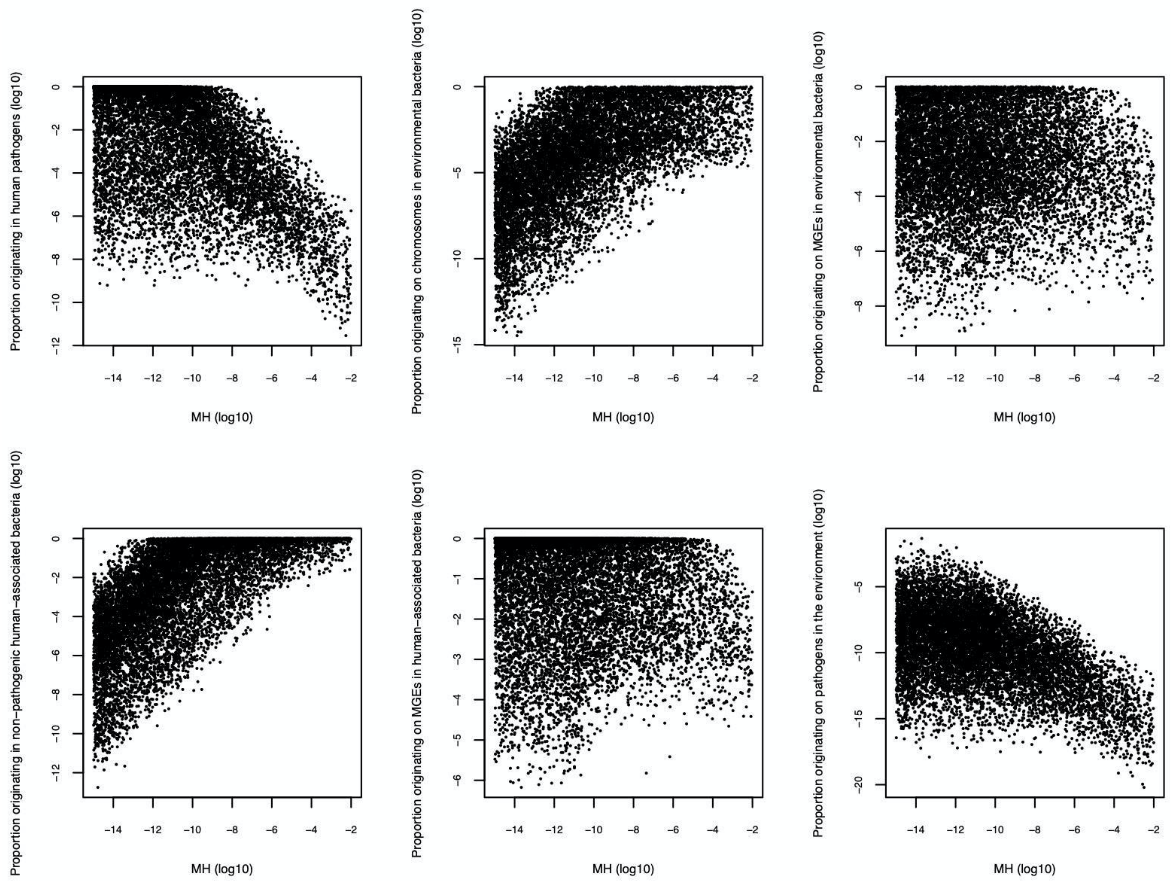

Supplementary Fig. S3. Dependency of the different processes on the MH parameter for the pre-existing model after 70 years of simulated time.

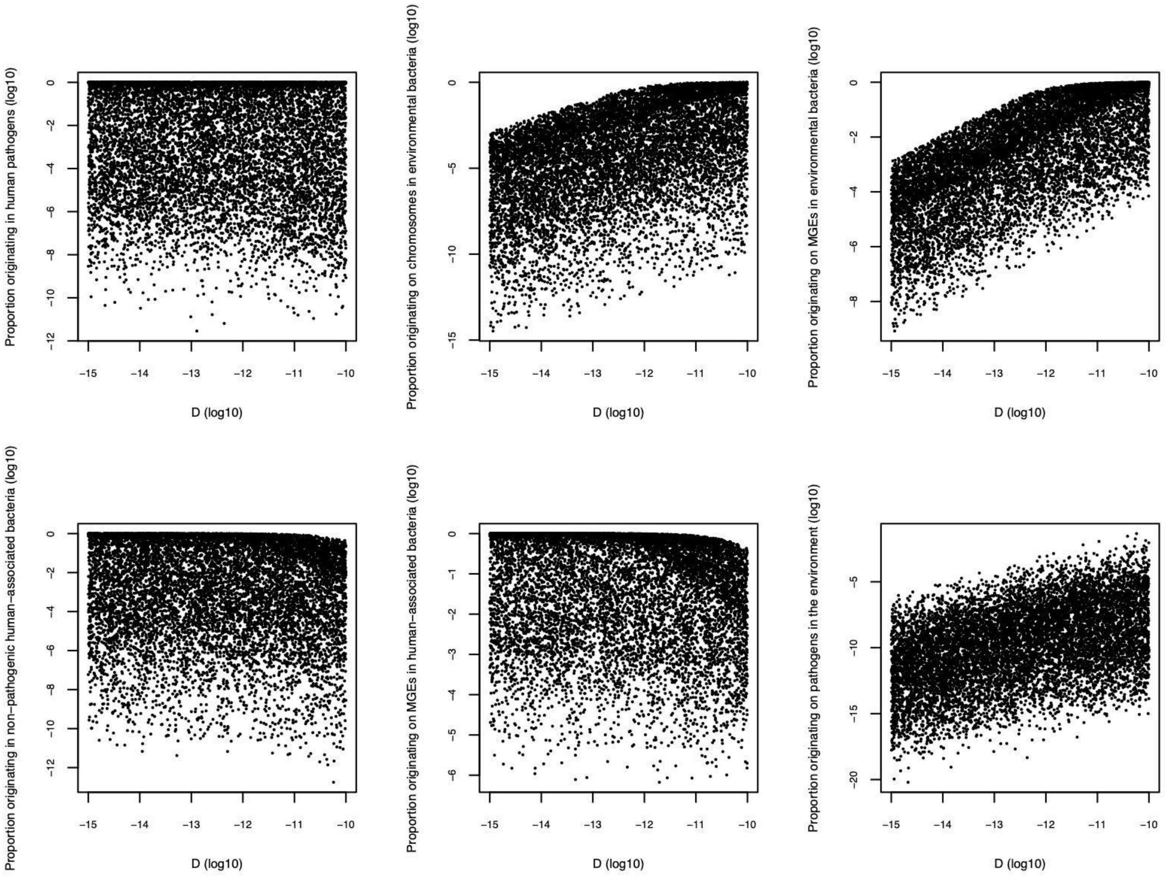

Supplementary Fig. S4. Dependency of the different processes on the D parameter for the pre-existing model after 70 years of simulated time.

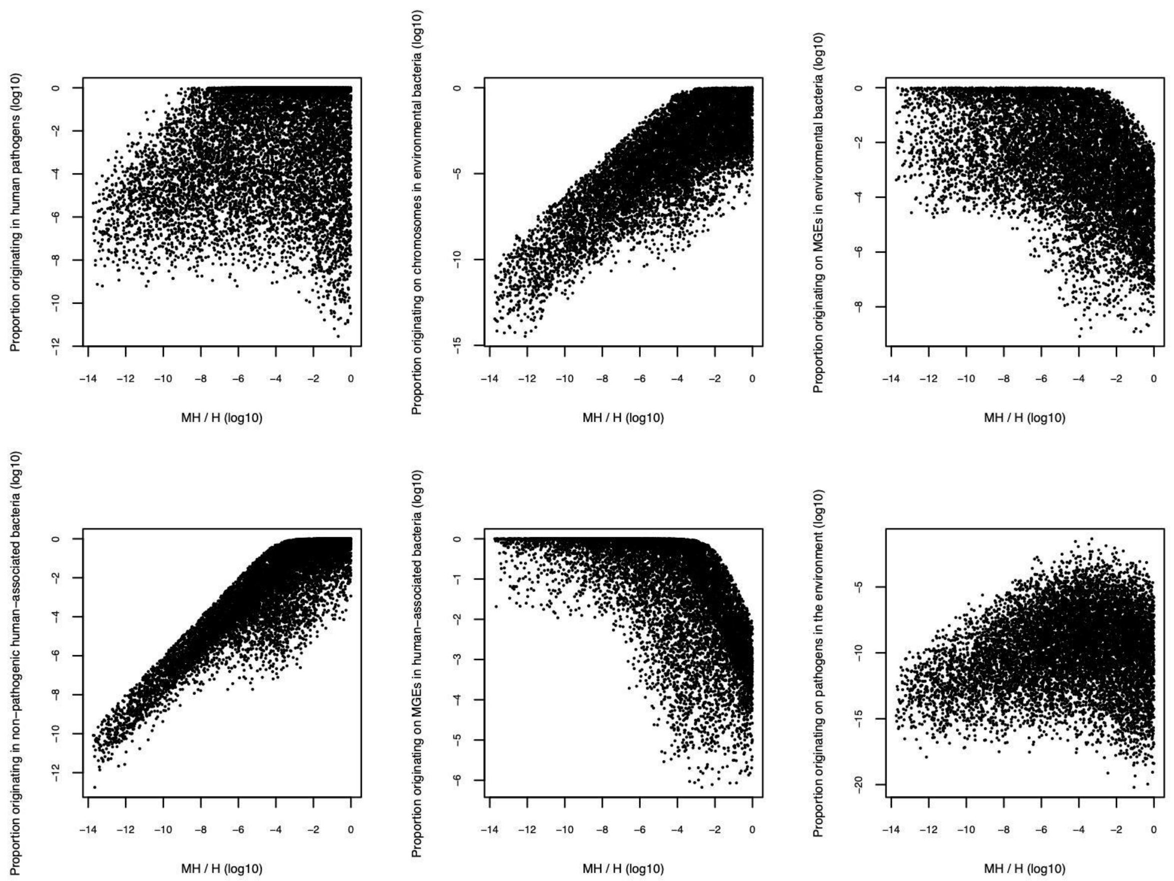

Supplementary Fig. S5. Dependency of the different processes on the MH parameter divided by the H parameter (resulting in an estimated M parameter) for the pre-existing model after 70 years of simulated time.

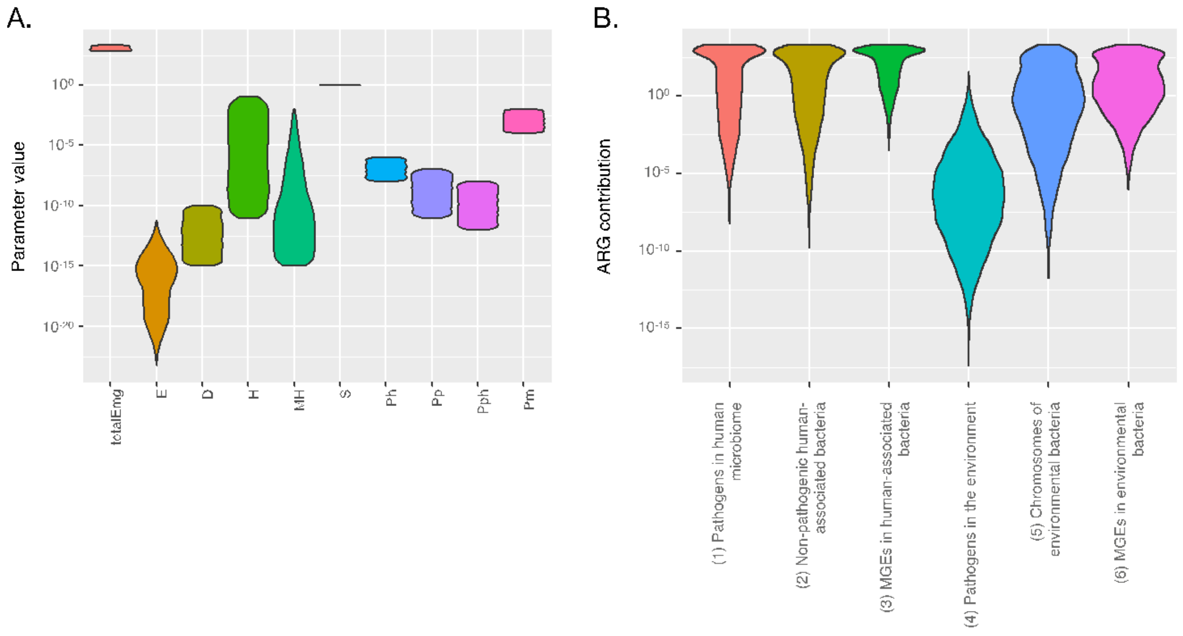

Supplementary Fig. S6. Valid parameter ranges (A) and process rates (B) for the pre-existing model after 70 years of simulated time with S being fixed to 1.

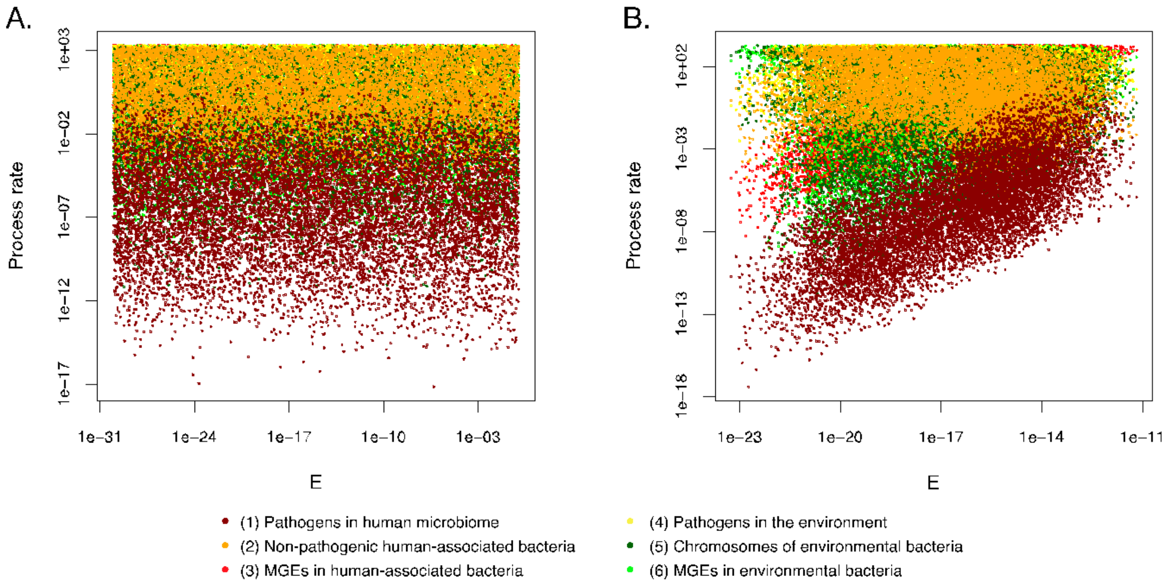

Supplementary Fig. S7. Differences between how processes depend on E for the pre-existing model (A), and the model where S is fixed to 1 (i.e. neutral fitness costs of ARGs, B). In both cases, data is presented at 70 years of simulated time.

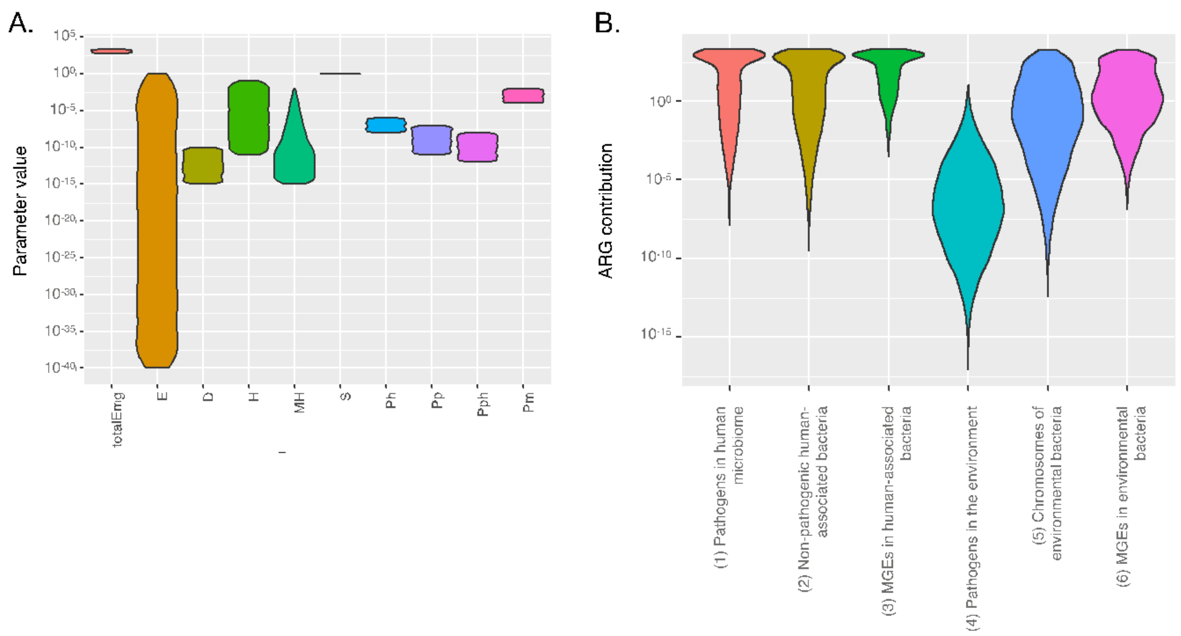

Supplementary Fig. S8. Valid parameter ranges (A) and process rates (B) for the emergence model after 70 years of simulated time.

Additional supplementary material

Code is available from here:
https://microbiology.se/publ/resistance_emergence_model/code.zip

Full results of the pre-existing (main) model:
https://microbiology.se/publ/resistance_emergence_model/preexisting_model.zip

Full results of the emergence model:
https://microbiology.se/publ/resistance_emergence_model/emergence_model.zip

References (supplement)

(1) Kusumaningrum, H. D.; Riboldi, G.; Hazeleger, W. C.; Beumer, R. R. Survival of Foodborne Pathogens on Stainless Steel Surfaces and Cross-Contamination to Foods. *Int J Food Microbiol* **2003**, *85* (3), 227–236. https://doi.org/10.1016/s0168-1605(02)00540-8.

(2) Bitton, G.; Farrah, S. R.; Ruskin, R. H.; Butner, J.; Chou, Y. J. Survival of Pathogenic and Indicator Organisms in Ground Water. *Groundwater* **1983**, *21* (4), 405–410. https://doi.org/10.1111/j.1745-6584.1983.tb00741.x.

(3) Lang, J. M.; Eisen, J. A.; Zivkovic, A. M. The Microbes We Eat: Abundance and Taxonomy of Microbes Consumed in a Day's Worth of Meals for Three Diet Types. *PeerJ* **2014**, *2*, e659. https://doi.org/10.7717/peerj.659.

(4) Bortolaia, V.; Kaas, R. S.; Ruppe, E.; Roberts, M. C.; Schwarz, S.; Cattoir, V.; Philippon, A.; Allesoe, R. L.; Rebelo, A. R.; Florensa, A. F.; Fagelhauer, L.; Chakraborty, T.; Neumann, B.; Werner, G.; Bender, J. K.; Stingl, K.; Nguyen, M.; Coppens, J.; Xavier, B. B.; Malhotra-Kumar, S.; Westh, H.; Pinholt, M.; Anjum, M. F.; Duggett, N. A.; Kempf, I.; Nykäsenoja, S.; Olkkola, S.; Wieczorek, K.; Amaro, A.; Clemente, L.; Mossong, J.; Losch, S.; Ragimbeau, C.; Lund, O.; Aarestrup, F. M. ResFinder 4.0 for Predictions of Phenotypes from Genotypes. *Journal of Antimicrobial Chemotherapy* **2020**, *75* (12), 3491–3500. https://doi.org/10.1093/jac/dkaa345.

(5) Crippen, C. S.; Jr., M. J. R.; Sanchez, S.; Szymanski, C. M. Multidrug Resistant Acinetobacter Isolates Release Resistance Determinants Through Contact-Dependent Killing and Bacteriophage Lysis. *Frontiers in Microbiology* **2020**, *11*. https://doi.org/10.3389/fmicb.2020.01918.

(6) Franke, A. E.; Clewell, D. B. Evidence for a Chromosome-Borne Resistance Transposon (Tn916) in Streptococcus Faecalis That Is Capable of "Conjugal" Transfer in the Absence of a Conjugative Plasmid. *Journal of Bacteriology* **1981**, *145* (1), 494–502. https://doi.org/10.1128/JB.145.1.494-502.1981.

(7) Scott, J. R.; Kirchman, P. A.; Caparon, M. G. An Intermediate in Transposition of the Conjugative Transposon Tn916. *Proceedings of the National Academy of Sciences of the United States of America* **1988**, *85* (13), 4809–4813.

(8) Torres, O. R.; Korman, R. Z.; Zahler, S. A.; Dunny, G. M. The Conjugative Transposon Tn925: Enhancement of Conjugal Transfer by Tetracycline in Enterococcus Faecalis and Mobilization of Chromosomal Genes in Bacillus Subtilis and E. Faecalis. *Molecular & general genetics : MGG* **1991**, *225* (3), 395–400.

(9) Poyart, C.; Celli, J.; Trieu-Cuot, P. Conjugative Transposition of Tn916-Related Elements from Enterococcus Faecalis to Escherichia Coli and Pseudomonas Fluorescens. *Antimicrobial Agents and Chemotherapy* **1995**, *39* (2), 500–506. https://doi.org/10.1128/AAC.39.2.500.

(10) Manson, J. M.; Hancock, L. E.; Gilmore, M. S. Mechanism of Chromosomal Transfer of Enterococcus Faecalis Pathogenicity Island, Capsule, Antimicrobial Resistance, and Other Traits. *Proceedings of the National Academy of Sciences of the United States of America* **2010**, *107* (27), 12269–12274. https://doi.org/10.1073/pnas.1000139107.

(11) Leonard, A. F. C.; Zhang, L.; Balfour, A. J.; Garside, R.; Gaze, W. H. Human Recreational Exposure to Antibiotic Resistant Bacteria in Coastal Bathing Waters. *Environment International* **2015**, *82*, 92–100. https://doi.org/10.1016/j.envint.2015.02.013.

(12) Leonard, A. F. C.; Zhang, L.; Balfour, A. J.; Garside, R.; Hawkey, P. M.; Murray, A. K.; Ukoumunne, O. C.; Gaze, W. H. Exposure to and Colonisation by Antibiotic-Resistant E. Coli in UK Coastal Water Users: Environmental Surveillance, Exposure Assessment, and Epidemiological Study (Beach Bum Survey). *Environment International* **2018**, *114*, 326–333. https://doi.org/10.1016/j.envint.2017.11.003.

(13) Sender, R.; Fuchs, S.; Milo, R. Revised Estimates for the Number of Human and Bacteria Cells in the Body. **2016**, *14* (8), e1002533. https://doi.org/10.1371/journal.pbio.1002533.

(14) Kallmeyer, J.; Pockalny, R.; Adhikari, R. R.; Smith, D. C.; D'Hondt, S. Global Distribution of Microbial Abundance and Biomass in Subseafloor Sediment. *Proceedings of the National Academy of Sciences of the United States of America* **2012**, *109* (40), 16213–16216. https://doi.org/10.1073/pnas.1203849109.

(15) Gregory, T. R. Synergy between Sequence and Size in Large-Scale Genomics. *Nat Rev Genet* **2005**, *6* (9), 699–708. https://doi.org/10.1038/nrg1674.

(16) Pal, C.; Bengtsson-Palme, J.; Kristiansson, E.; Larsson, D. G. J. Co-Occurrence of Resistance Genes to Antibiotics, Biocides and Metals Reveals Novel Insights into Their Co-Selection Potential. *BMC Genomics* **2015**, *16* (1), 964. https://doi.org/10.1186/s12864-015-2153-5.

(17) Hausner, M.; Wuertz, S. High Rates of Conjugation in Bacterial Biofilms as Determined by Quantitative in Situ Analysis. *Applied and Environmental Microbiology* **1999**, *65* (8), 3710–3713.

(18) Wan, Z.; Varshavsky, J.; Teegala, S.; McLawrence, J.; Goddard, N. L. Measuring the Rate of Conjugal Plasmid Transfer in a Bacterial Population Using Quantitative PCR. *Biophys J* **2011**, *101* (1), 237–244. https://doi.org/10.1016/j.bpj.2011.04.054.

(19) Simonsen, L.; Gordon, D. M.; Stewart, F. M.; Levin, B. R. Estimating the Rate of Plasmid Transfer: An End-Point Method. *J Gen Microbiol* **1990**, *136* (11), 2319–2325. https://doi.org/10.1099/00221287-136-11-2319.

(20) Andrup, L.; Andersen, K. A Comparison of the Kinetics of Plasmid Transfer in the Conjugation Systems Encoded by the F Plasmid from Escherichia Coli and Plasmid PCF10 from Enterococcus Faecalis. *Microbiology (Reading)* **1999**, *145 (Pt 8)*, 2001–2009. https://doi.org/10.1099/13500872-145-8-2001.

(21) Zhong, X.; Droesch, J.; Fox, R.; Top, E. M.; Krone, S. M. On the Meaning and Estimation of Plasmid Transfer Rates for Surface-Associated and Well-Mixed Bacterial Populations. *J Theor Biol* **2012**, *294*, 144–152. https://doi.org/10.1016/j.jtbi.2011.10.034.
(22) Jutkina, J.; Rutgersson, C.; Flach, C.-F.; Larsson, D. G. J. An Assay for Determining Minimal Concentrations of Antibiotics That Drive Horizontal Transfer of Resistance. *The Science of the total environment* **2016**, *548–549*, 131–138. https://doi.org/10.1016/j.scitotenv.2016.01.044.
(23) Normander, B.; Christensen, B. B.; Molin, S.; Kroer, N. Effect of Bacterial Distribution and Activity on Conjugal Gene Transfer on the Phylloplane of the Bush Bean (Phaseolus Vulgaris). *Applied and Environmental Microbiology* **1998**, *64* (5), 1902–1909.
(24) Suhartono, S.; Savin, M. Conjugative Transmission of Antibiotic-Resistance from Stream Water Escherichia Coli as Related to Number of Sulfamethoxazole but Not Class 1 and 2 Integrase Genes. *Mob Genet Elements* **2016**, *6* (6), e1256851. https://doi.org/10.1080/2159256X.2016.1256851.
